# FoxO3 normalizes Smad3-induced arterial smooth muscle cell growth

**DOI:** 10.3389/fphys.2023.1136998

**Published:** 2023-08-24

**Authors:** Jake T. Francisco, Andrew W. Holt, Michael T. Bullock, Madison D. Williams, Cere E. Poovey, Nathan A. Holland, Jeffrey J. Brault, David A. Tulis

**Affiliations:** Department of Physiology, Brody School of Medicine, East Carolina University, Greenville, NC, United States

**Keywords:** adenoviral overexpression, arterial smooth muscle, cardiovascular disease, FoxO3, MuRF-1, phosphorylation, Smad3

## Abstract

Transition of arterial smooth muscle (ASM) from a quiescent, contractile state to a growth-promoting state is a hallmark of cardiovascular disease (CVD), a leading cause of death and disability in the United States and worldwide. While many individual signals have been identified as important mechanisms in this phenotypic conversion, the combined impact of the transcription factors Smad3 and FoxO3 in ASM growth is not known. The purpose of this study was to determine that a coordinated, phosphorylation-specific relationship exists between Smad3 and FoxO3 in the control of ASM cell growth. Using a rat *in vivo* arterial injury model and rat primary ASM cell lysates and fractions, validated low and high serum *in vitro* models of respective quiescent and growth states, and adenoviral (Ad-) gene delivery for overexpression (OE) of individual and combined Smad3 and/or FoxO3, we hypothesized that FoxO3 can moderate Smad3-induced ASM cell growth. Key findings revealed unique cellular distribution of Smad3 and FoxO3 under growth conditions, with induction of both nuclear and cytosolic Smad3 yet primarily cytosolic FoxO3; Ad-Smad3 OE leading to cytosolic and nuclear expression of phosphorylated and total Smad3, with almost complete reversal of each with Ad-FoxO3 co-infection in quiescent and growth conditions; Ad-FoxO3 OE leading to enhanced cytosolic expression of phosphorylated and total FoxO3, both reduced with Ad-Smad3 co-infection in quiescent and growth conditions; Ad-FoxO3 inducing expression and activity of the ubiquitin ligase MuRF-1, which was reversed with concomitant Ad-Smad3 OE; and combined Smad3/FoxO3 OE reversing both the pro-growth impact of singular Smad3 and the cytostatic impact of singular FoxO3. A primary takeaway from these observations is the capacity of FoxO3 to reverse growth-promoting effects of Smad3 in ASM cells. Additional findings lend support for reciprocal antagonism of Smad3 on FoxO3-induced cytostasis, and these effects are dependent upon discrete phosphorylation states and cellular localization and involve MuRF-1 in the control of ASM cell growth. Lastly, results showing capacity of FoxO3 to normalize Smad3-induced ASM cell growth largely support our hypothesis, and overall findings provide evidence for utility of Smad3 and/or FoxO3 as potential therapeutic targets against abnormal ASM growth in the context of CVD.

## 1 Introduction

Cardiovascular disease (CVD) persists as the major cause of illness and death in the United States (American Heart Association, 2023) and across the globe (World Health Organization, 2021). While major advances have been made in our understanding of many causes of CVD, its pervasiveness continues to escalate, thereby demanding expanded efforts to identify new genetic, molecular, and/or cellular underpinnings of CVD in hopes of highlighting potential new therapeutic targets.

Of the many cell types in cardiovascular tissues implicated in CVD pathology, arterial smooth muscle (ASM) is of primary importance (Tulis, 2017; Holland et al., 2018). Healthy mature ASM cells display a differentiated and contractile phenotype, allowing the essential functions of vessel contraction and dilation while maintaining low levels of proliferation in a quiescent steady state. However, following stimulation from growth factors, mitogens and/or inflammatory mediators during disease or injury, ASM cells convert to a proliferative phenotype with enhanced protein synthesis and growth and with loss of contractile elements. We’ve detailed various aspects of phenotypic transformation in ASM cell growth in the context of disease or injury (Holt et al., 2016; Holland et al., 2018; Williams et al., 2023). In brief, cell growth is multifactorial, involving alterations in DNA ploidy, cell size (hypertrophy), cell number (hyperplasia), cell movement (chemotaxis; directed/non-directed migration), and/or matrix balance. These various forms of phenotypic change from a quiescent to a growth state diminish normal vessel function and contribute to clinical manifestations including alterations in local blood pressures, vessel wall remodeling and neointimal development, and lumen stenosis with ischemia and hypoxia of downstream tissues, central elements of CVD (Holt et al., 2016; Tulis, 2017; Holland et al., 2018). Reasonably, approaches aimed at controlling phenotypic transformation and pathologic ASM growth are of significant scientific and clinical interest.

In conversion to a growth phenotype, ASM cells become increasingly sensitive to growth and mitogenic factors such as transforming growth factor-β (TGF-β), Erk1/2 (p44/p42 MAPK), interleukins, TNF-α, PDGF, and angiotensin II. These factors contribute to the pathogenesis of CVD through their capacities to provoke abnormal matrix synthesis, cell proliferation, and cell migration and chemotaxis, all central elements in CVD pathogenesis (Adderley et al., 2012; Holland et al., 2018). A factor of significant scientific inquiry over the past several decades, TGF-β is a pluripotent cytokine that can impact cellular processes such as migration, proliferation, differentiation, recognition, adhesion, and apoptosis, all in context-specific and sometimes paradoxical fashion (Shi and Massague, 2003). Following receptor activation, TGF-β can utilize Smad and/or non-Smad pathways to carry out its functions (Shi and Massague, 2003; Javelaud and Mauviel, 2004), yet its primary intracellular effector in cardiovascular tissues is receptor-regulated Smad3. Previous studies from our lab (Tulis et al., 2005; Stone et al., 2015), using recombinant TGF-β delivery and adenovirus (Ad)-mediated Smad3 overexpression (OE), support a role for TGF-β-driven Smad3 in promoting a growth phenotype in ASM. Complementary findings show that TGF-β-induced Smad3 operates through Erk1/2 signaling (Suwanabol et al., 2012), likely involving nuclear export and inactivation of the cell cycle inhibitor p27 (Tsai et al., 2009) to promote ASM growth. Other findings from our group (Bollinger et al., 2014) suggest that Smad3 operates in a dynamic and cooperative fashion with the transcription factor Forkhead box O3 (FoxO3) in mediating downstream events. Intriguingly, FoxO3 has been implicated as growth inhibitory (Mahajan et al., 2012; Tucka et al., 2014), operating in part through induction of cytostatic p21 and/or p27 (Abid et al., 2005; Mahajan et al., 2012; Yu et al., 2018), but its relationship with Smad3 as combined transcriptional regulators in ASM is not known. Further, cellular signaling and overall biological activities of Smad3 and FoxO3 are governed, at least in part, by ubiquitin ligases specific for Smad3, such as Smurf-1/-2 (Zhang et al., 2001; Piersma et al., 2015; Koganti et al., 2018), and FoxO3, such as MuRF-1 and Atrogin-1 (Sandri et al., 2004; Cohen et al., 2009; Demasi and Laurindo, 2012; Paffett et al., 2012; Bollinger et al., 2014). Ubiquitin ligases are implicated in cell cycle control in growth pathologies (Dang et al., 2021), and Atrogin-1 and MuRF-1 are both involved in smooth muscle atrophy and loss and uterine involution (Bdolah et al., 2007). Lastly, Smad3 and FoxO3 act in opposition in terms of their phosphorylation-dependent transcriptional activation: Smad3 phosphorylation at Ser_423/425_ is necessary for translocation into the nucleus where activated Smad3 can exert its transcriptional effects (Shi and Massague, 2003; Javelaud and Mauviel, 2004), while FoxO3 phosphorylation at Thr_32_ prevents translocation into the nucleus and results in cytosolic retention and inactivation (Gomis et al., 2006; Fu and Peng, 2011). As such, several states of transcriptional activation for Smad3 and FoxO3 exist based on phosphorylation status and cellular localization. Our understanding of a Smad3/FoxO3 association in ASM is limited, particularly with respect to their unique phosphorylation profiles and particularly in the setting of ASM cell growth, and more in-depth study is warranted.

The purpose of this study was to determine that a coordinated, phosphorylation-specific relationship exists between the transcription factors Smad3 and FoxO3 in the control of ASM cell growth. Using a rat arterial injury model and rat primary ASM cell lysates and fractions, low and high serum conditions as respective models of quiescent and growth states, and Ad-gene delivery for OE of individual and combined Smad3 and FoxO3, key findings reveal cellular distribution of Smad3 and FoxO3 during growth, with induction of both nuclear and cytosolic Smad3 yet primarily cytosolic FoxO3; Ad-Smad3 OE leading to robust cytosolic and nuclear expression of phosphorylated and total Smad3, with almost complete reversal of each with Ad-FoxO3 co-infection in both quiescent and growth conditions; Ad-FoxO3 OE leading to enhanced cytosolic expression of phosphorylated and total FoxO3, both reduced with concomitant Ad-Smad3 in both quiescent and growth conditions; Ad-FoxO3 inducing expression and activity of the ubiquitin ligase MuRF-1, which was reversed with Ad-Smad3 co-infection; and combined Smad3/FoxO3 OE reversing both the pro-growth impact of Smad3 and the cytostatic impact of FoxO3 in ASM cells. These observations lend evidence for an antagonistic relationship between the transcription factors Smad3 and FoxO3 with emphasis on their discrete phosphorylation states and cellular localization and with involvement of MuRF-1 in the control of ASM cell growth. These findings also provide support for their utility as potential therapeutic targets in ASM growth control in the context of CVD.

## 2 Materials and methods

### 2.1 Animals

All experimental procedures involving animals strictly abided by guidelines of the East Carolina University Animal Care and Use Committee and the Guide for the Care and Use of Laboratory Animals (National Research Council, 2011). Animal experiments were designed according to ARRIVE 2.0 guidelines (Percie du Sert et al., 2020). Only male rats (CD^®^ Sprague-Dawley IGS; strain 001; Charles River) were used to offset potential influence of female hormones on ASM growth as we’ve documented (Yuan et al., 2002). Animals were housed in 12-h light/dark cycles with food and water provided *ad libitum*.

### 2.2 Rat carotid artery balloon injury

Following our protocols (Tulis, 2007a; Tulis, 2007b; Holt and Tulis, 2013), adult rats (425–475 g body weight) were anesthetized (ketamine (90 mg/mL), xylazine (10 mg/mL); 1 mL/kg, intraperitoneal injection) and provided analgesia (Buprenex; 0.5 mL/kg, subcutaneous injection) supplied by the Department of Comparative Medicine, ECU. The left carotid artery and external carotid branch were surgically exposed, and a Fogarty 2FR embolectomy catheter (Baxter Healthcare Corp., Irvine, CA) was introduced into an external carotid arteriotomy and advanced to the aortic arch. The balloon was inflated to 2 kPa and was withdrawn three times with rotation to remove the endothelium and mechanically distend the vessel wall. The catheter was removed, the external carotid artery was ligated both proximal and distal to the arteriotomy, vessel patency and common carotid blood flow were verified, and overlying tissues were closed in layers. Thirty minutes after injury (a time point appropriate to detect changes in phosphorylation events), animals were euthanized, and injured and uninjured (naïve) carotid arteries were harvested for Western blot evaluation of Smad3 and FoxO3.

### 2.3 Rat primary ASM cell culture

As routine in our lab (Tulis et al., 2003; Mendelev et al., 2009; Joshi et al., 2011; Holt et al., 2016; Williams et al., 2023), juvenile rats (100–150 g body weight) were anesthetized and euthanized via pneumothorax and exsanguination. Thoracic aortae were surgically removed, mechanically and chemically (collagenase, elastase) digested, and incubated in a bead bath at 37° for 4 h. Tissues were centrifuged at 400 rcf for 5 min at room temperature (RT). Pelleted tissue was resuspended in 5 mL of Dulbecco’s Modified Eagle Medium (DMEM) (ThermoFisher) supplemented with 10% fetal bovine serum (Gemini Bio-Products) and 0.2% Primocin (InvivoGen) and seeded into T12 tissue culture (TC) vented flasks. Derived primary ASM cells were incubated in 5% CO_2_ at 37° untouched for 72 h to allow for adherence and spreading. Cell confluence was verified using a Leica DMI4000B inverted microscope. ASM cell makeup was periodically determined by Western blotting for the ASM-specific marker SM22α (Li et al., 1996; Williams et al., 2023), and cells were serially passaged (P) up to P6 to prevent onset of phenotypic switching (Boerth et al., 1997; Williams et al., 2023).

### 2.4 Adenovirus infection

Following our protocols for adenoviral gene delivery (Tulis et al., 2001; Tulis et al., 2003; Stone et al., 2015), cells were seeded in 12-well TC-treated plates at a density of 100,000 cells/well until adherent. Conditioned media (CM) was aspirated, and cells were quiesced (DMEM with 0.2% FBS, 0.2% Primocin) for 24 h, after which CM was removed and cells were infected with 1 mL of CM containing either adenovirus (Ad) incorporating full-length green fluorescent protein (GFP; 1:10,000), Smad3, FoxO3, or Smad3/FoxO3 (1:3,000 each) for 24 h. Of note, all Ad-cohorts except Ad-Smad3 contain an internal ribosome entry site (IRES) that precedes the GFP coding sequence. Following infection, Ad-containing media was aspirated, cells were washed, and 1 mL of either CM or quiescent media was added for an additional 24, 48, or 72 h per experimental conditions. Representative images of Ad-infected ASM cells were captured using a Leica DMI4000B inverted microscope.

### 2.5 Protein isolation and detection

Following treatment, CM was aspirated and ASM cells were trypsinized, centrifuged at 500 rcf, and washed with ice-cold DPBS. Cells were lysed using ice-cold RIPA buffer (ThermoFisher) supplemented with Halt protease and phosphatase inhibitor cocktail (1:100; ThermoFisher). Cell lysates were centrifuged at 14,000 rcf for 15 min at 4°C, and protein concentrations were determined using Coomassie (Bradford) Protein Assay Kit (ThermoFisher). Cell lysates (or fractions, see below) were subjected to sodium dodecyl sulfate polyacrylamide gel electrophoresis (SDS-PAGE) with stain-free gels (Bio-Rad). Separated protein was transferred onto polyvinylidene fluoride (PVDF) membranes (Bio-Rad) with Trans-Blot Turbo RTA Transfer Kit (Bio-Rad) using Trans-Blot Turbo Transfer System (Bio-Rad) using the mixed molecular weight setting (7 min at 2.5 A and 25 V). Following transfer, total protein was obtained using ChemiDoc MP Imaging System (Bio-Rad), and membranes were blocked for 1 h at RT in 5% dry milk solution in 0.1% Tris-buffered saline with Tween-20 (TBST). Membranes were subjected to three 10-min washes in TBST and incubated overnight at 4°C in 5% bovine serum albumin (BSA) in 0.1% TBST using the following primary antibodies at 1:500–1:1,000 dilution per manufacturers’ guidelines: Smad3 (#9513; Cell Signaling Technology); Smad3-Ser_423/425_ (#9520; Cell Signaling Technology); FoxO3 (#2497; Cell Signaling Technology); FoxO3-Thr_32_ (#ab26649; Abcam); Erk1/2 (#4695; Cell Signaling Technology); Erk1/2-Thr_202_/Tyr_204_ (#9101; Cell Signaling Technology); Atrogin-1 (#AP 2041; ECM Biosciences); MuRF-1 (#AF5366; bio-techne); Smurf-1 (#2714; Cell Signaling Technology); Smurf-2 (#12024; Cell Signaling Technology); p21 Waf1/Cip1 (#2947; Cell Signaling Technology); p27 Kip1 (#3688; Cell Signaling Technology); GAPDH (#2118; Cell Signaling Technology); lamin A/C (#612621; BD Transduction Laboratories); PKG-1 (#3248; Cell Signaling Technology). The following day, membranes were washed three times for 10 min each and incubated at RT for 1–2 h using 1:10,000 horseradish peroxidase (HRP)-conjugated anti-rabbit secondary antibodies (Rockland) or an anti-goat secondary antibody (for MuRF-1). Membranes were then washed three times for 10 min each and developed using West Pico substrates (ThermoFisher) in 1:1 dilution. Enhanced chemiluminescence (ECL) was utilized for protein detection with the ChemiDoc MP Imaging System. Protein was quantified using ImageLab v6.1 software (Bio-Rad) and normalized to total protein from the same sample and from the same gel as recently recommended best practice for rigorous normalization of sample loading variabilities in Western blotting (Brooks and Lindsey, 2018).

### 2.6 Cell fractionation

Cells were harvested at 48- or 72-h post-infection, and a Subcellular Protein Fractionation Kit for Cultured Cells (ThermoFisher) was used per the manufacturer’s instructions to obtain cytoplasmic and nuclear fractions. Fractionated samples were subjected to Western blot analyses for select protein targets including the fraction-specific proteins lamin A/C (nuclear) and GAPDH (cytoplasmic). Cytoplasmic or nuclear target proteins were normalized to respective total cytoplasmic protein or total nuclear protein as appropriate (Brooks and Lindsey, 2018).

### 2.7 Luciferase reporter assays

Following our protocol (Bollinger et al., 2014), ASM cells were washed, collected in 1x passive lysis buffer, centrifuged (20,000 g for 5 min), and 20 µL supernatant was pipetted into wells containing 100 µL substrate in white 96-well plates per manufacturer’s instructions (Promega). Luminescence was measured in a microplate reader (SpectraMax M4, Molecular Devices), total protein per sample was determined using a BCA protein assay (ThermoScientific), and luminescence values were normalized to total protein and expressed as fold-change relative to Ad-GFP (control) cells.

### 2.8 Flow cytometry and cell cycle progression

Flow cytometry was performed as routine in our lab (Stone et al., 2012; Holt et al., 2016). Following treatment (24, 48, or 72 h), CM was aspirated, and cells were trypsinized, centrifuged at 500 g for 5 min, rinsed with PBS, centrifuged for 5 min, stained with FxCycle propidium iodide (PI)/RNase Staining Solution (Invitrogen), subjected to flow cytometry (BD FACScan), and analyzed with ModFit LT software to estimate number/percentage of cells within each cell cycle phase.

### 2.9 Cell viability, proliferation, and attachment assays

Following treatment, CM was aspirated, cells were trypsinized, and cell numbers were determined through automated quantification with trypan blue exclusion staining (ViCell, Beckman Coulter) to provide objective measures of viability and total and viable cell numbers as we’ve detailed (Joshi et al., 2011; Stone et al., 2015; Holt et al., 2016; Williams et al., 2023). Cell attachment was determined using a crystal violet assay (ab232855; Abcam) per manufacturer’s instructions with microplate analysis read at 570 nm absorbance.

### 2.10 Immunofluorescence and confocal microscopy

Cells were quiesced (0.2% FBS) for 24 h, maintained in either 0.2% (low) serum or switched to 10% (high) serum for 24 h, then washed twice with RT DPBS, fixed in 4% paraformaldehyde for 15 min at RT, and blocked (1X DPBS, 5% FBS, 0.3% Triton™ X-100) for 60 min. Blocking buffer was aspirated and primary antibody in buffer (1:500 for both Smad3 and FoxO3; in 1X PBS, 1% BSA, 0.3% Triton™ X-100) was added and incubated overnight at 4°C. Cells were washed twice with RT DPBS, and fluorochrome-conjugated secondary antibody buffer added for 1–2 h at RT. Representative images were captured on Zeiss LSM710 confocal microscope.

### 2.11 Statistical analyses

Data management and statistical analysis was performed using Prism 10 (GraphPad Software, LLC), with data presented as mean ± standard error of the mean (SEM). Statistical significance between multiple cohorts within a particular experiment was determined using a one-way analysis of variance (ANOVA) or a repeated measures two-way ANOVA with assessment of interaction effects. When the *F* ratio indicated a significant change occurred, Tukey’s test or a multiple replicate Bonferroni *post hoc* test was performed to identify individual paired differences. Two group comparisons were analyzed using a paired Student’s t-test. Sample sizes varied per experiment and are shown in the appropriate Figure Legends. Statistical significance was determined by a *p*-value <0.05.

## 3 Results

### 3.1 *In vivo* arterial injury induces Smad3

**FIGURE 1:**
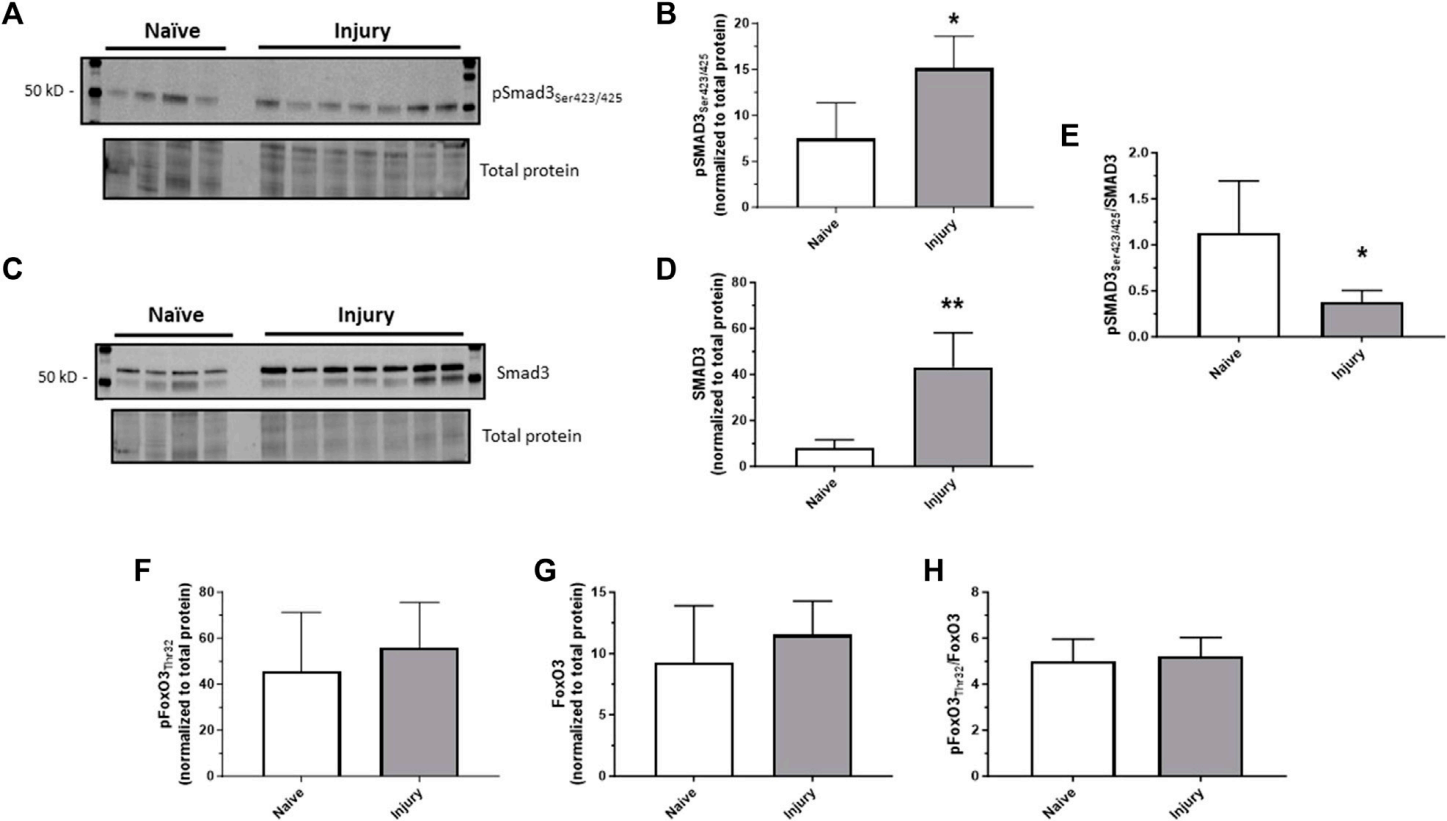
*In vivo arterial injury induces Smad3* Rat left carotid arteries were balloon injured, harvested after 30 min, and Western blotting was performed on arterial homogenates from injured and naïve (uninjured) samples for phosphorylated Smad3 (Ser_423/425_); **(A,B)**, total Smad3 **(C,D)**, phosphorylated FoxO3 (Thr_32_), and total FoxO3. Densitometry data (normalized to total cellular protein from the same blot) show significantly increased phosphorylated **(B)** and total **(D)** Smad3 in injured arteries compared to control arteries. The percent increase in total Smad3 exceeded the percent increase in phosphorylated Smad3, so the phosphorylated to total Smad3 ratio was significantly reduced in injured arteries compared to controls **(E)**. Neither phosphorylated **(F)** nor total **(G)** FoxO3, nor their ratio **(H)**, was significantly altered in injured arteries compared to control arteries. *p < 0.05, **p < 0.01 vs. naïve controls; *n* = 4 rats/naïve group; *n* = 7 rats/injured group.

Considering our previous observations that TGF-β significantly increased in injured carotid arteries and that TGF-β was (at least partly) required for ensuing arterial growth and remodeling (Tulis et al., 2005), new experiments were designed to discern Smad3 as a primary effector of TGF-β in injured vessels using rat carotid artery balloon injury with assessment of Smad3 expression and activity. Homogenates of injured arteries harvested 30 min post-injury demonstrated significantly increased levels of both phosphorylated (Ser_423/425_) Smad3 and total Smad3 compared to naïve uninjured controls (Figures 1A–D). As the percent increase in total Smad3 exceeded the percent increase in phosphorylated Smad3, the phospho- to total Smad3 ratio was significantly reduced in injured arteries compared to controls (Figure 1E). Arterial balloon injury did not significantly affect expression of phosphorylated (Thr_32_) FoxO3 (Figure 1F), total FoxO3 (Figure 1G), or phospho/total FoxO3 (Figure 1H) compared to controls. These observations in intact arteries provided rationale to specifically examine Smad3, with consideration of possible influence by FoxO3, during growth and paved the way for a more precisely controlled, *in vitro* study using a clonal, homogenous ASM cell population outside of the heterogenous, multi-cellular environment of the *in vivo* setting.

### 3.2 Validation of low serum and high serum media as respective models of quiescent and growth states

**FIGURE 2:**
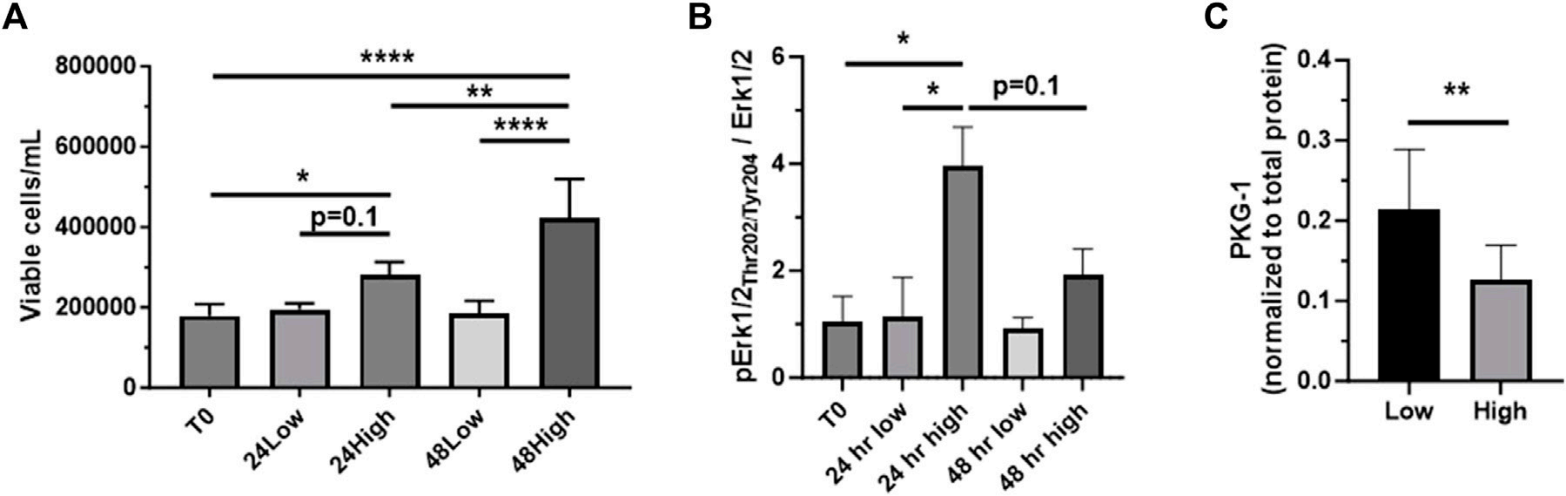
*Confirmation of low serum/high serum in vitro models* Rat primary ASM cells were quiesced (0.2% serum) for 24 h and then incubated in low serum (0.2%) or high serum (10%) media through 48 h, after which total and viable cell numbers were obtained. Shown in **(A)**, viable ASM cell numbers significantly increased in high serum media after both 24 and 48 h compared to that at time 0 (T0). Also, cells in high serum were markedly elevated (*p* = 0.1) after 24 h and significantly elevated after 48 h compared to cells in low serum. **(B)** Cell homogenates were then prepared after 24 and 48 h and probed for phosphorylated (Thr_202_/Tyr_204_) and total Erk1/2. The phosphorylated Erk1/2 to total Erk1/2 ratio was significantly increased in cells in high serum versus those in low serum after 24 h. Interestingly, this increase was largely diminished after 48 h, still showing a non-significant doubling of the phosphorylated Erk1/2 to total Erk1/2 ratio at that time point. **(C)** Evaluation of PKG-1 expression as an established marker of phenotypic switching. High serum-treated ASM cells showed significantly reduced PKG-1 expression compared to cells under low serum after 48 h. *n* = 12 well/cohort; *p < 0.05, **p < 0.01, ****p < 0.0001 vs. controls for comparisons as shown.

To confirm our use of low serum (0.2%) and high serum (10%) media as respective *in vitro* models of quiescent and growth states, ASM cells were grown in low or high serum media through 48 h, after which viable cell counts were measured. Seen in Figure 2A, after 24 h a non-significant increase (*p* = 0.1) in viable cell numbers in high serum media compared to low serum media was observed, with this difference reaching significance after 48 h. In these samples we probed for phosphorylated (Thr_202_/Tyr_204_) and total Erk1/2 as known mitogenic agents (Roskoski Jr, 2012; Suwanabol et al., 2012). After 24 h, cells grown in high serum media displayed a significantly increased phosphorylated to total Erk1/2 ratio compared to cells grown in low serum media (Figure 2B). Interestingly, this difference was largely gone after 48 h, shown by a non-significant doubling of the phospho/total Erk1/2 ratio in cells incubated in high serum. Next, we probed 48 h low serum- and high serum-treated cells for PKG-1, an established marker of phenotypic switching (Boerth et al., 1997; Williams et al., 2023). As seen in Figure 2C, PKG-1 expression was significantly reduced in cells incubated in high serum media versus low serum media, consistent with our recent findings in ASM cells under growth-stimulating conditions (Williams et al., 2023).

### 3.3 Cellular distribution of Smad3 and FoxO3 during growth

**FIGURE 3:**
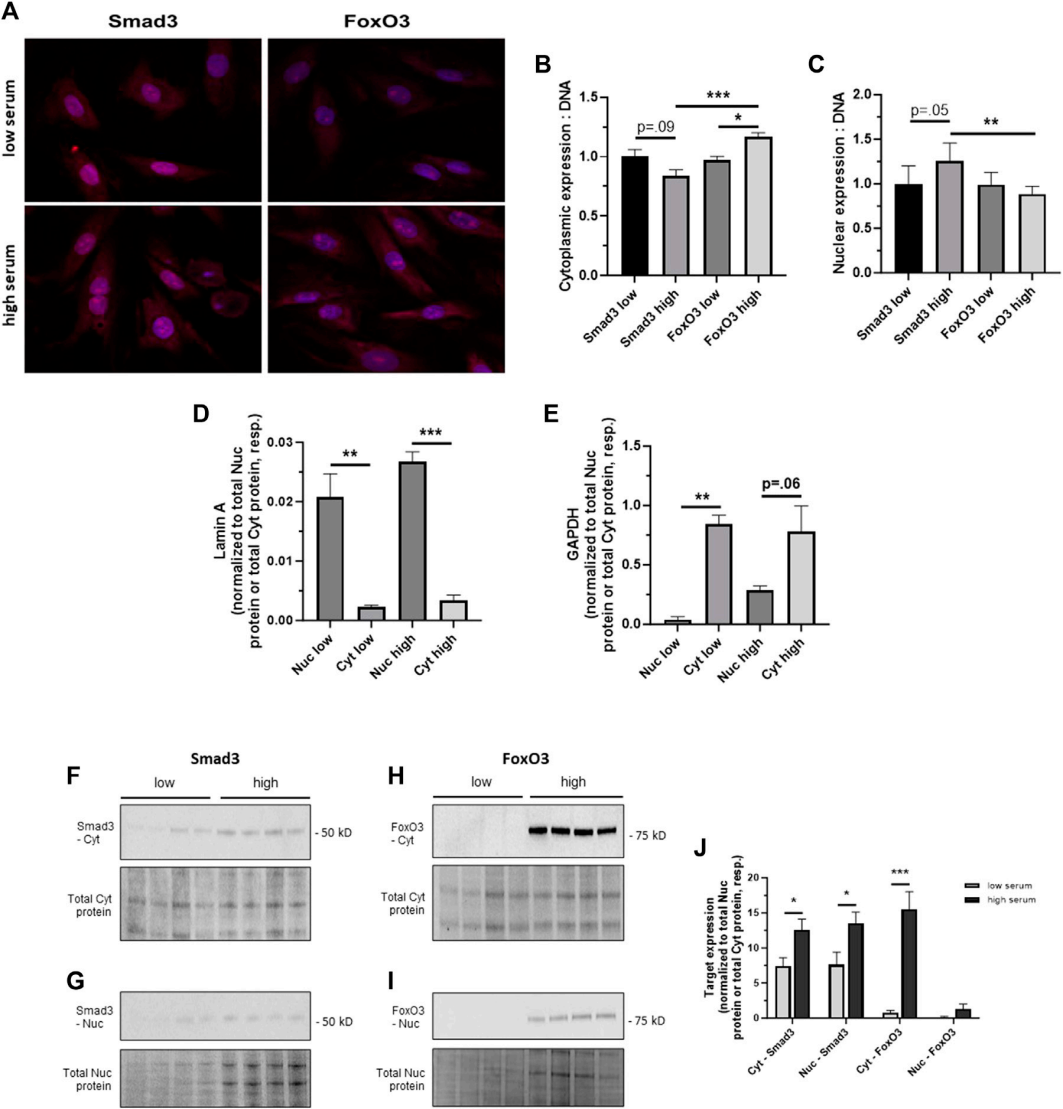
*Cellular distribution of Smad3 and FoxO3 during growth* **(A)** Confocal photomicrographs of rat adherent ASM cells immunostained for Smad3 or FoxO3 under low (0.2%) serum, quiescent conditions or high (10%) serum, growth-stimulated conditions for 24 h. Densitometry data normalized to cellular DNA content suggest trends for decreased cytoplasmic Smad3 (*p* = 0.09) and increased nuclear Smad3 (*p* = 0.05) in high serum compared to low serum **(B,C)**. In comparison, image densitometry suggests significantly increased cytoplasmic FoxO3 in high serum conditions compared to low serum conditions **(B)**. Further, in high serum media, expression of cytoplasmic FoxO3 was significantly higher than that of cytoplasmic Smad3, and expression of nuclear FoxO3 was significantly lower than that of nuclear Smad3. *n* = 3 independent experiments/cohort. **(D,E)** Validation of nuclear (Nuc) and cytoplasmic (Cyt) cell fractions. Rat primary ASM cells were incubated in low (0.2%) or high (10%) serum for 24 h, after which protein expression of lamin A and GAPDH was determined. Western blot densitometry revealed that lamin A was significantly expressed in the nucleus compared to the cytoplasm (each respectively normalized to total nuclear protein or total cytoplasmic protein) under both low and high serum conditions **(D)**. In comparison, GADPH was significantly expressed in the cytoplasm in low serum conditions and was markedly higher (*p* = 0.06) in high serum conditions compared to expression in the nucleus **(E)**, again each normalized to total nuclear protein or total cytoplasmic protein. **(F–J)** Following 24 h in low or high serum, rat ASM cells were homogenized, cell fractions were generated, and Western blots were performed for cytoplasmic and nuclear Smad3 and FoxO3 expression. Representative blots are shown for Cyt Smad3 **(F)**, Nuc Smad3 **(G)**, Cyt FoxO3 **(H)**, and Nuc FoxO3 **(I)**, each with a total cytoplasmic protein or total nuclear protein blot, respectively, from the same gel. In **(J)**, densitometry revealed significantly increased cytoplasmic and nuclear Smad3 (each normalized to total cytoplasmic or nuclear protein, as appropriate, from the same blot) in high serum versus low serum as well as significantly increased cytoplasmic FoxO3 (normalized to total cytoplasmic protein from the same blot) in high serum versus low serum, without significant changes in nuclear FoxO3 expression under these conditions. *n* = 4 independent experiments/cohort; *p < 0.05, **p < 0.01, ***p < 0.001 for comparisons as shown.

Rat primary ASM cells were incubated in low (0.2%) or high (10%) serum to mimic quiescent and growth conditions, respectively, as we’ve described (Tulis et al., 2003; Joshi et al., 2011; Stone et al., 2012; Stone et al., 2015). Confocal images of Smad3- and FoxO3-immunostained adherent cells captured after 24 h in low or high serum are shown in Figure 3A. Densitometry performed on the confocal images suggests that Smad3 expression was markedly (*p* = 0.09) lower in the cytoplasm and markedly higher (*p* = 0.05) in the nucleus in high serum compared to low serum media (Figures 3B, C). In comparison, image densitometry suggests that cytoplasmic FoxO3 expression was significantly elevated in high versus low serum media (Figure 3B). Further, in high serum media, cytoplasmic FoxO3 was significantly increased over cytoplasmic Smad3 (Figure 3B), and nuclear FoxO3 was significantly lower than nuclear Smad3 (Figure 3C). To address subjectivity and the lack of precise quantitation associated with performing image densitometry and to more accurately determine cellular localization of Smad3 and FoxO3 during growth, ASM cells were incubated in low or high serum media for 24 h, after which cytoplasmic and nuclear fractions were generated and probed for lamin A and GAPDH (for validation of cellular fractions) as well as for Smad3 and FoxO3 protein expression. Shown in Figure 3D, lamin A expression was significantly greater in the nucleus (normalized to total nuclear protein) compared to the cytoplasm (normalized to total cytoplasmic protein) under both low and high serum conditions. In comparison, GADPH expression was significantly greater in the cytoplasm in low serum conditions, and markedly higher (*p* = 0.06) in high serum conditions (both normalized to total cytoplasmic protein), compared to expression in the nucleus (normalized to total nuclear protein; Figure 3E). Western blotting for cytoplasmic and nuclear Smad3 and FoxO3 was then performed in cells in low and high serum media (Figures 3F–I). Densitometry of the Western blots (Figure 3J) revealed significantly increased cytoplasmic and nuclear Smad3 (each normalized to total cytoplasmic or total nuclear protein, respectively, from the same blot) in high serum versus low serum. Similarly, significantly increased cytoplasmic FoxO3 (normalized to total cytoplasmic protein from the same blot) in high serum versus low serum was observed, yet without significant changes in nuclear FoxO3 under these conditions.

### 3.4 Ad-mediated OE of Smad3, FoxO3

**FIGURE 4:**
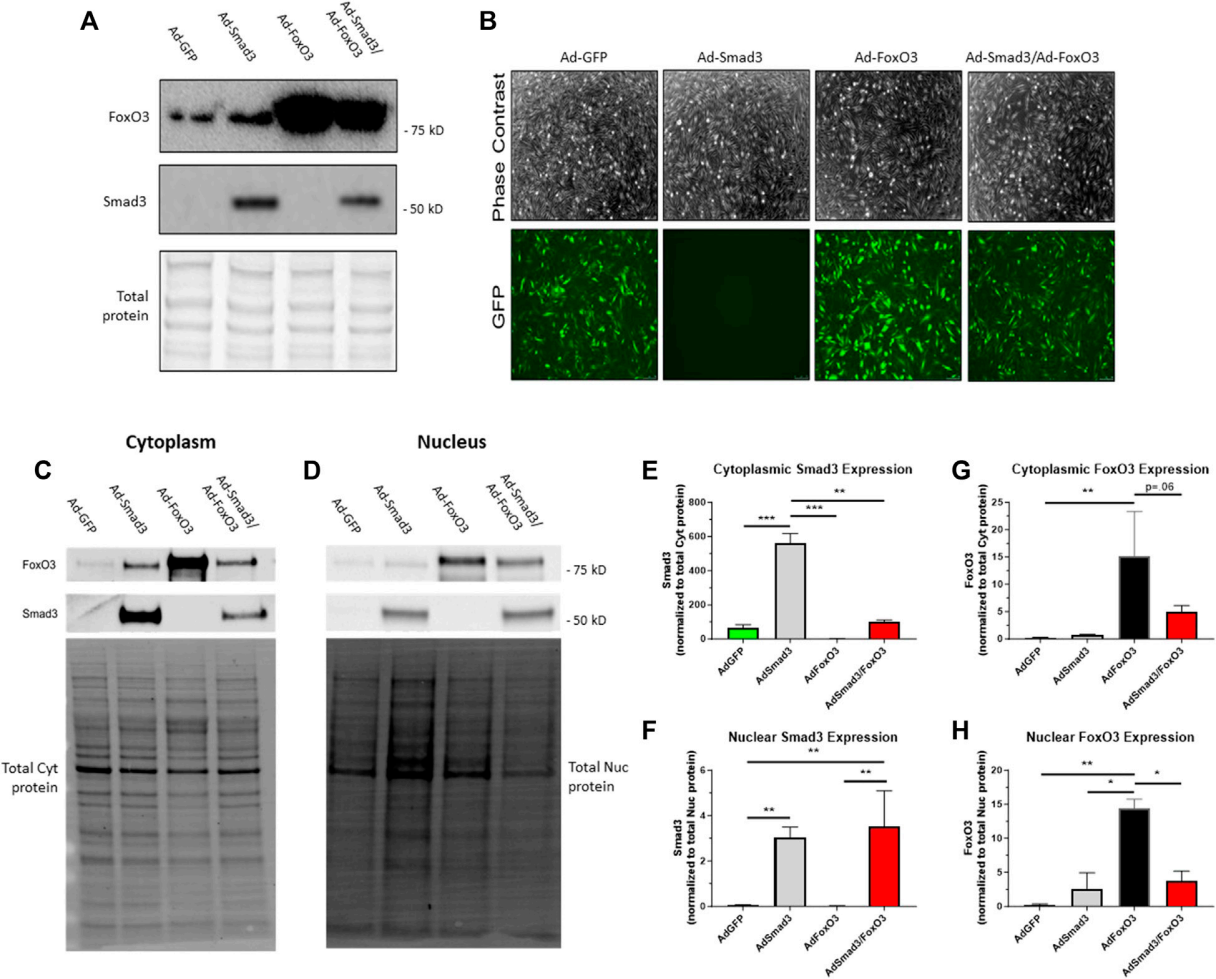
*Ad-mediated OE of Smad3, FoxO3* Rat ASM cells were quiesced (0.2% serum) for 24 h and then infected with Ad-GFP, Ad-Smad3 (does not encode GFP), Ad-FoxO3, or Ad-Smad3/Ad-FoxO3 for 24 h, after which Ad-was removed, cells were washed, high (10%) serum was added, and cells were carried out for 24–72 h. **(A)** A representative Western blot for Smad3 and FoxO3 along with a total cellular protein blot (from the same gel) on Ad-infected ASM cell lysates 24 h post-infection is shown. **(B)** Phase contrast and GFP fluorescent photomicrographs of Ad-infected ASM cells after 72 h are shown (Ad-Smad3 does not encode GFP). To determine cellular localization of Smad3 and FoxO3 following Ad- OE, cytoplasmic and nuclear fractions from Ad-infected cells were generated after 48 h and were probed for Smad3 and FoxO3. Representative Western blots for cytoplasmic and nuclear Smad3 and FoxO3 along with total cytoplasmic protein and total nuclear protein blots from the same gels are shown **(C,D)**. Densitometry reveals significantly increased cytoplasmic Smad3 in Ad-Smad3 cells, which was significantly reduced in Ad-Smad3/Ad-FoxO3 cells **(E)**. Likewise, nuclear Smad3 was significantly elevated with Ad-Smad3 infection compared to Ad-GFP cells, yet this did not change with Ad-Smad3/Ad-FoxO3 OE **(F)**. Cytoplasmic FoxO3 significantly increased versus Ad-GFP cells following Ad-FoxO3 infection, and this was largely reduced (*p* = 0.06) in Ad-Smad3/Ad-FoxO3 cells **(G)**. Nuclear FoxO3 was significantly increased in Ad-FoxO3 cells compared to Ad-GFP controls, and this was significantly reversed in Ad-Smad3/Ad-FoxO3 cells **(H)**. *n* = 3 independent experiments/group; *p < 0.05, **p < 0.01, ***p < 0.001 for comparisons as shown.

We have previously optimized targeted Ad-mediated gene delivery to ASM, an approach that bypasses potential confounding influence of side effects from upstream agonists, for discrete OE of desired targets (Tulis et al., 2001; Tulis et al., 2003) including Smad3 (Stone et al., 2015). In the current study, using GFP fluorescent intensity measurements (for Ad-GFP, Ad-FoxO3, and Ad-Smad3/Ad-FoxO3 cells, all containing IRES elements that precede the GFP coding sequence), we determined Ad-infection efficiencies between 55% and 65% between 24 and 72 h (data not shown). Using a Smad-binding element luciferase (SBE-luc) reporter assay to measure Smad-mediated transcriptional activity (Bollinger et al., 2014) for Ad-Smad3 and Ad-Smad3/Ad-FoxO3 cells, we validated Smad-dependent transcriptional activities for Ad-Smad3 (15-fold higher than Ad-GFP) and Ad-Smad3/Ad-FoxO3 (30-fold higher than Ad-GFP) groups (data not shown). Following establishment of the efficiencies of our Ad-infections and validation of increased transcriptional activities following OE of Smad3 and FoxO3, Western blotting for Smad3 and FoxO3 was performed on Ad-infected whole ASM cell lysates 24 h post-infection. Figure 4A shows a sample blot for Smad3 and FoxO3 along with a blot for total cellular protein from the same gel as a loading control. Phase contrast and GFP fluorescent photomicrographs of Ad-infected adherent ASM cells taken 72 h post-infection (Figure 4B) illustrate consistent levels of Ad-infection across all groups (keeping in mind that Ad-Smad3 does not contain IRES that encodes GFP, so Ad-Smad3 is void of GFP fluorescence). Further, to determine cellular localization of Smad3 and FoxO3 following Ad- OE, ASM cells were infected for 24 h, and 48 h later cytoplasmic and nuclear fractions were probed for Smad3 and FoxO3. Representative Western blots for cytoplasmic and nuclear Smad3 and FoxO3 along with total cytoplasmic protein and total nuclear protein blots, respectively, from the same gels are shown in Figures 4C, D. Densitometry revealed significantly increased cytoplasmic Smad3 in Ad-Smad3 cells, which was significantly reduced in the Ad-Smad3/Ad-FoxO3 group (Figure 4E). Similarly, nuclear Smad3 was significantly elevated with Ad-Smad3 infection, but this did not change with Ad-Smad3/Ad-FoxO3 OE (Figure 4F). Cytoplasmic FoxO3 was significantly increased compared to Ad-GFP controls following Ad-FoxO3 infection, and this was mostly reduced (*p* = 0.06) in Ad-Smad3/Ad-FoxO3 cells (Figure 4G). Nuclear FoxO3 was significantly increased in Ad-FoxO3 cells, which again returned to baseline levels in Ad-Smad3/Ad-FoxO3 cells (Figure 4H).

### 3.5 Cellular localization of phosphorylated and total Smad3 and FoxO3 following Ad- OE during quiescent and growth conditions

**FIGURE 5:**
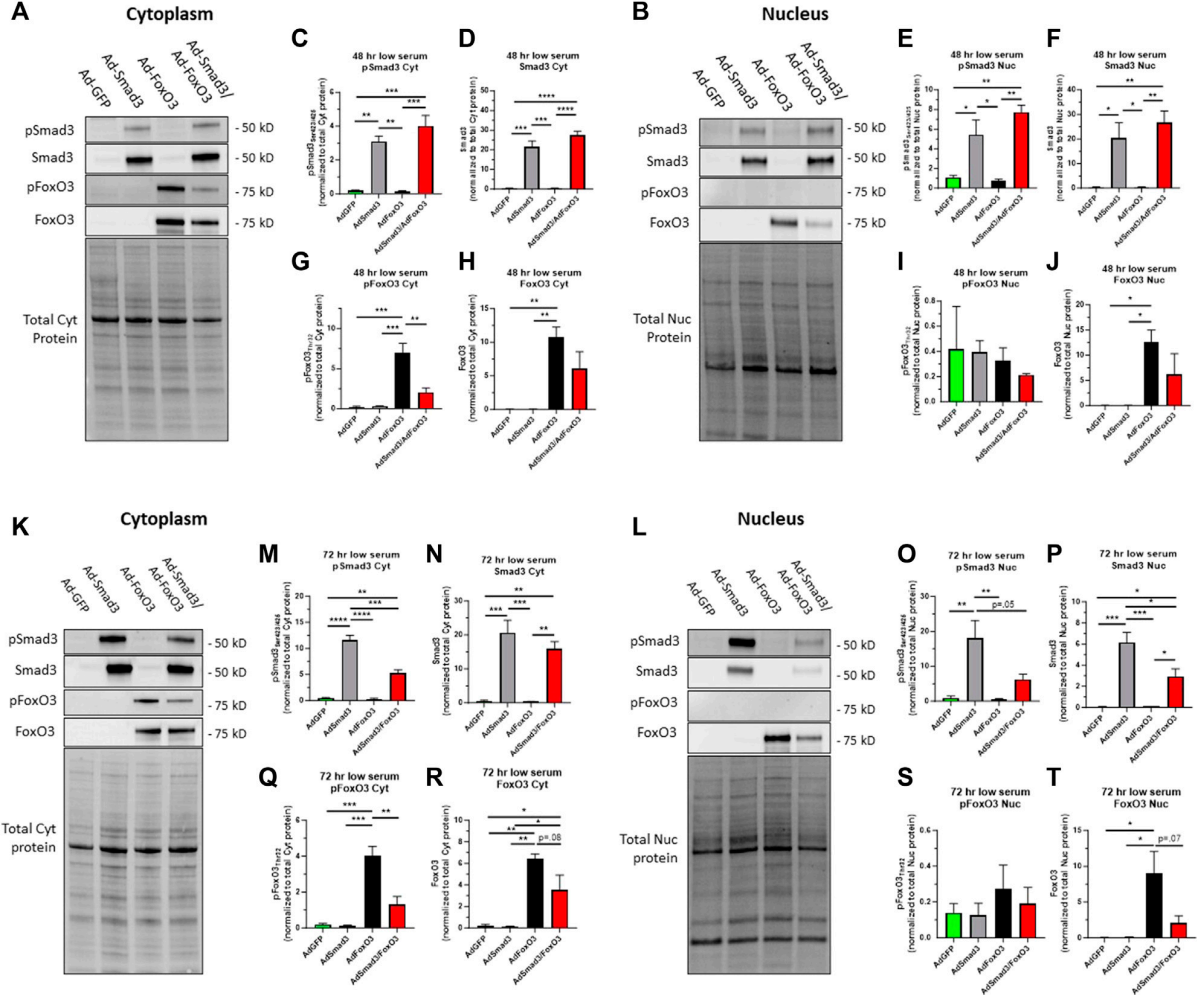
*Cellular localization of phosphorylated and total Smad3 and FoxO3 following Ad- OE in quiescent cells* Rat ASM cells were infected for 24 h with Ad-GFP, Ad-Smad3, Ad-FoxO3, or Ad-Smad3/Ad-FoxO3, and then grown in low (0.2%) serum quiescent conditions for 48 h **(A–J)** or 72 h **(K–T)**, after which cytoplasmic and nuclear fractions were probed for phosphorylated and total Smad3 and FoxO3. Representative Western blots for cytoplasmic **(A)** and nuclear **(B)** phosphorylated and total Smad3 and phosphorylated and total FoxO3 along with total cytoplasmic protein and total nuclear protein blots, respectively, from the same gels after 48 h in quiescent, low serum media are shown. Densitometry revealed that Ad-Smad3 cells grown in low serum showed significant expression of phosphorylated and total Smad3 in the cytoplasm **(C,D)** and nucleus **(E,F)** compared to Ad-GFP cells. Concomitant Ad-Smad3/Ad-FoxO3 OE did not significantly alter phosphorylated or total Smad3 expression observed with Ad-Smad3 alone in the cytoplasm **(C,D)** or nucleus **(E,F)** at 48 h. Under these same quiescent conditions for 48 h, FoxO3-infected cells showed significantly increased cytoplasmic phosphorylated and total FoxO3 **(G,H)**, and with Ad-Smad3/Ad-FoxO3 OE these levels were significantly reduced for phosphorylated FoxO3 **(G)** with noticeable (∼50%; n.s.) reduction in total FoxO3 **(H)**. Further, in the nucleus, FoxO3 OE did not impact phosphorylated FoxO3 levels **(I)**, but significantly increased total FoxO3 **(J)** which similarly was reduced (∼50%; n.s.) in Ad-Smad3/Ad-FoxO3 cells **(J)**. Of note, absolute levels of phosphorylated FoxO3 in the nucleus **(I)** were barely detectable compared to absolute levels of phosphorylated FoxO3 in the cytoplasm **(G)** or total FoxO3 in the cytoplasm **(H)** or nucleus **(J)**. Representative Western blots for cytoplasmic **(K)** and nuclear **(L)** phosphorylated and total Smad3 and phosphorylated and total FoxO3 along with total cytoplasmic protein and total nuclear protein blots, respectively, from the same gels after 72 h in quiescent, low serum media are shown. Densitometry reveals that Ad-Smad3 cells grown in low serum showed significant expression of phosphorylated and total Smad3 in the cytoplasm **(M,N)** and nucleus **(O,P)** compared to Ad-GFP cells. Concomitant Ad-Smad3/Ad-FoxO3 infection significantly reversed cytoplasmic phosphorylated Smad3 **(M)**, markedly (*p* = .05) reduced nuclear phosphorylated Smad3 **(O)**, and significantly reduced nuclear total Smad3 **(P)** compared to Ad-Smad3 OE alone. Under these same quiescent conditions, FoxO3-infected cells showed significantly increased cytoplasmic phosphorylated and total FoxO3 **(Q,R)**, and these levels were significantly reduced for phosphorylated FoxO3 **(Q)** and markedly (∼50%; *p* = 0.08) reduced for total FoxO3 **(R)** with Ad-Smad3/Ad-FoxO3 OE. Further, in the nucleus, FoxO3 OE did not noticeably impact phosphorylated FoxO3 levels **(S)**, but significantly increased total FoxO3 **(T)**, and similarly, nuclear total FoxO3 was markedly reduced (*p* = 0.07) in Ad-Smad3/Ad-FoxO3 cells **(T)**. Of note, absolute levels of phosphorylated FoxO3 in the nucleus **(S)** were barely detectable compared to absolute levels of phosphorylated FoxO3 in the cytoplasm **(Q)** or total FoxO3 in the cytoplasm **(R)** or nucleus **(T)**. *n* = 4 independent experiments/group; *p < 0.05, **p < 0.01, ***p < 0.001, ****p < 0.0001 for comparisons as shown.

Site-specific phosphorylation of both Smad3 and FoxO3 is essential for their activation: phosphorylation of Smad3 at Ser_423/425_ results in activation in the nucleus while phosphorylation of FoxO3 at Thr_32_ leads to nuclear export and cytoplasmic localization and inactivation (Yang and Hung, 2009; Fu and Peng, 2011; Deng et al., 2015). As such, Smad3 and FoxO3 act in opposition in terms of phosphorylation-dependent transcriptional activation. To determine phosphorylation status and therefore transcriptional activities of Smad3 and FoxO3 under quiescent or growth conditions, ASM cells were infected for 24 h with Ad-GFP, Ad-Smad3, Ad-FoxO3, or Ad-Smad3/Ad-FoxO3, and then grown in either low (0.2%) serum or high (10%) serum media, respective models of quiescence or growth, for 48 or 72 h, after which cytoplasmic and nuclear fractions were probed for phosphorylated and total Smad3 and FoxO3 (each normalized to total cytoplasmic protein or total nuclear protein, respectively). Figures 5A, B show respective Western blots for cytoplasmic and nuclear phosphorylated and total Smad3 and FoxO3 along with respective total cytoplasmic protein and total nuclear protein blots from the same gels after 48 h in low serum quiescent media. At this time point, Ad-Smad3-infected ASM cells grown in low serum showed significant expression of phosphorylated and total Smad3 in both the cytoplasm (Figures 5C, D) and nucleus (Figures 5E, F) compared to Ad-GFP cells. Concomitant Ad-Smad3/Ad-FoxO3 infection failed to noticeably affect these expression patterns for Smad3 in both the cytoplasm and nucleus. Under these same low serum conditions, FoxO3-infected cells showed significantly increased cytoplasmic phosphorylated and total FoxO3 (Figures 5G, H), with cytoplasmic phosphorylated FoxO3 being significantly reversed with Ad-Smad3/Ad-FoxO3 infection (Figure 5G). In the nucleus, FoxO3 OE did not noticeably impact phosphorylated FoxO3 levels (Figure 5I), but significantly increased total FoxO3 (Figure 5J) compared to GFP cells. Of note, absolute levels of phosphorylated FoxO3 in the nucleus (Figure 5I) were very low relative to absolute levels of phosphorylated or total FoxO3 in the cytoplasm (Figures 5G, H) or total FoxO3 in the nucleus (Figure 5J).

In comparison, using this same experimental plan with low serum quiescent media but with assessment after 72 h, respective Western blots for cytoplasmic and nuclear phosphorylated and total Smad3 and FoxO3 with respective total cytoplasmic protein and total nuclear protein blots from the same gels in low serum media are shown (Figures 5K, L). At this time point, Ad-Smad3-infected ASM cells grown in low serum continued to show significant expression of phosphorylated and total Smad3 in both the cytoplasm (Figures 5M, N) and nucleus (Figures 5O, P) compared to Ad-GFP cells. Interestingly, concomitant Ad-Smad3/Ad-FoxO3 infection significantly reversed cytoplasmic phosphorylated Smad3 (Figure 5M), largely (*p* = 0.05) reduced nuclear phosphorylated Smad3 (Figure 5O), and significantly reduced nuclear total Smad3 (Figure 5P) compared to Ad-Smad3 infection alone. Under these same quiescent low serum conditions, FoxO3-infected cells continued to show significantly increased cytoplasmic phosphorylated and total FoxO3 (Figures 5Q, R), and these levels were significantly reduced for phosphorylated FoxO3 and markedly (∼50%; *p* = 0.08) reduced for total FoxO3 with Ad-Smad3/Ad-FoxO3 infection. In the nucleus, FoxO3 OE again did not noticeably impact phosphorylated FoxO3 levels (Figure 5S), but again significantly increased total FoxO3 (Figure 5T) compared to GFP cells, with nuclear total FoxO3 levels being largely reduced (*p* = 0.07) in the Ad-Smad3/Ad-FoxO3 cells (Figure 5T). Similar to that observed after 48 h, at the 72-h time point absolute levels of phosphorylated FoxO3 in the nucleus (Figure 5S) were very low relative to absolute levels of phosphorylated or total FoxO3 in the cytoplasm (Figures 5Q, R) or total FoxO3 in the nucleus (Figure 5T).

**FIGURE 6:**
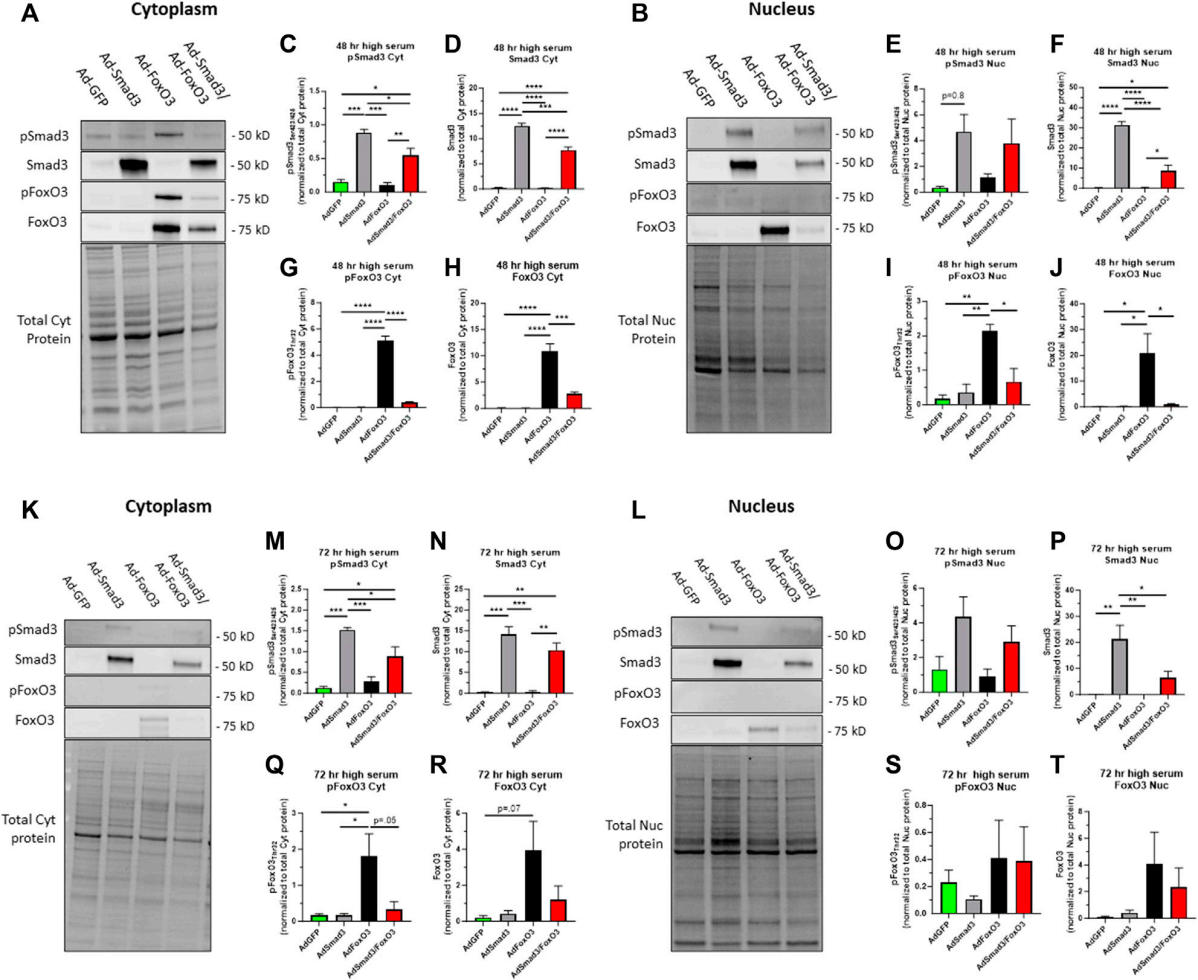
*Cellular localization of phosphorylated and total Smad3 and FoxO3 following Ad- OE during growth* Rat ASM cells were infected for 24 h with Ad-GFP, Ad-Smad3, Ad-FoxO3, or Ad-Smad3/Ad-FoxO3, and then grown in high (10%) serum, growth media for 48 h **(A–J)** or 72 h **(K–T)**, after which cytoplasmic and nuclear fractions were probed for phosphorylated and total Smad3 and FoxO3. Representative Western blots for cytoplasmic **(A)** and nuclear **(B)** phosphorylated and total Smad3 and phosphorylated and total FoxO3 along with total cytoplasmic protein and total nuclear protein blots, respectively, from the same gels after 48 h in high serum growth media are shown. Densitometry reveals that Smad3 OE cells in the Ad-Smad3 and Ad-Smad3/Ad-FoxO3 groups showed significant increases in phosphorylated and total Smad3 in the cytoplasm **(C,D)**, with a trend for increased phosphorylated Smad3 (*p* = 0.8; **(E)** and significantly increased total Smad3 in the nucleus **(F)**, compared to Ad-GFP cells. Interestingly, the Ad-Smad3/Ad-FoxO3 cells showed significantly reduced phosphorylated and total Smad3 in the cytoplasm **(C,D)** and significantly reduced total Smad3 in the nucleus **(F)** compared to Ad-Smad3 cells alone. Under these growth conditions at 48 h, Ad-FoxO3-infected cells showed significantly increased cytoplasmic phosphorylated and total FoxO3 **(G,H)** compared to Ad-GFP cells. Further, these increases were fully and significantly reversed with concomitant Ad-Smad3/Ad-FoxO3 infection **(G,H)**. In the nucleus, Ad-FoxO3-infected cells showed significantly increased phosphorylated and total FoxO3 **(I,J)** compared to Ad-GFP cells, and FoxO3 expression was significantly reversed with Ad-Smad3/Ad-FoxO3 OE **(I,J)**. Absolute levels of phosphorylated FoxO3 in the nucleus **(I)** were very low compared to levels of phosphorylated or total FoxO3 in the cytoplasm **(G,H)** or total nuclear FoxO3 **(J)**. Representative Western blots for cytoplasmic **(K)** and nuclear **(L)** phosphorylated and total Smad3 and phosphorylated and total FoxO3 along with total cytoplasmic protein and total nuclear protein blots, respectively, from the same gels after 72 h in high serum growth media are shown. Densitometry reveals that Smad3 OE cells in the Ad-Smad3 and Ad-Smad3/Ad-FoxO3 groups showed significant increases in phosphorylated and total Smad3 in the cytoplasm **(M,N)** as well as increased total Smad3 in the nucleus **(O, P)** compared to Ad-GFP cells. Interestingly, the Ad-Smad3/Ad-FoxO3 cells showed significantly reduced phosphorylated Smad3 in the cytoplasm **(M)** and significantly reduced total Smad3 in the nucleus **(P)** compared to Ad-Smad3 cells alone. Under these growth conditions, Ad-FoxO3-infected cells showed significantly increased cytoplasmic phosphorylated FoxO3 **(Q)** with a trend (*p* = 0.07) for increased cytoplasmic total FoxO3 **(R)**, compared to Ad-GFP cells. Further, these increases were largely reversed with concomitant Ad-Smad3/Ad-FoxO3 infection **(Q,R)**. No significant changes in phosphorylated or total FoxO3 expression were detected in the nucleus across all Ad-groups **(S,T)**. Absolute levels of phosphorylated FoxO3 in the nucleus **(S)** were relatively very low compared to levels of phosphorylated or total FoxO3 in the cytoplasm **(Q,R)** or total nuclear FoxO3 **(T)**. *n* = 4 independent experiments/group; *p < 0.05, **p < 0.01, ***p < 0.001, ****p < 0.0001 for comparisons as shown.

Next, in growth-promoting high serum (10%) media, cells were infected with Ad-GFP, Ad-Smad3, Ad-FoxO3, or Ad-Smad3/Ad-FoxO3 for 48 and 72 h, after which cells were fractionated and probed for Smad3 and FoxO3 expression. Figures 6A, B show respective Western blots for cytoplasmic and nuclear phosphorylated and total Smad3 and cytoplasmic and nuclear phosphorylated and total FoxO3 along with total cytoplasmic protein and total nuclear protein blots, respectively, from the same gels. Smad3-infected cells in the Ad-Smad3 and Ad-Smad3/Ad-FoxO3 groups showed significant increases in phosphorylated and total Smad3 in the cytoplasm (Figures 6C, D), a marked trend (*p* = 0.08) for increased phosphorylated Smad3 in the nucleus (Figure 6E), and significantly increased total Smad3 in the nucleus (Figure 6F) compared to Ad-GFP cells. Ad-Smad3/Ad-FoxO3 cells showed significantly reduced phosphorylated and total Smad3 in the cytoplasm (Figures 6C, D) and significantly reduced total Smad3 in the nucleus (Figure 6F) compared to the Ad-Smad3 groups alone. Also, under these high serum, growth conditions, Ad-FoxO3-infected cells showed significantly increased cytoplasmic phosphorylated and total FoxO3 (Figures 6G, H) as well as significantly increased nuclear phosphorylated and total FoxO3 (Figures 6I, J) compared to Ad-GFP controls. Further, these increases in FoxO3 were all significantly reversed with concomitant Ad-Smad3/Ad-FoxO3 infection (Figures 6G–J). Absolute levels of phosphorylated FoxO3 in the nucleus (Figure 6I) were extremely low compared to absolute levels of phosphorylated or total cytoplasmic FoxO3 (Figures 6G, H) or total nuclear FoxO3 (Figure 6J).

In comparison, using this same experimental plan using high serum growth media but with assessment after 72 h, respective Western blots for cytoplasmic and nuclear phosphorylated and total Smad3 and FoxO3 with respective total cytoplasmic protein and total nuclear protein blots from the same gels in low serum media are shown (Figures 6K, L). Seen in Figures 6M, N, Smad3 OE cells in the Ad-Smad3 and Ad-Smad3/Ad-FoxO3 groups showed significant increases in phosphorylated and total Smad3 in the cytoplasm as well as increased total Smad3 in the nucleus (Figure 6P) compared to Ad-GFP cells. Again, the Ad-Smad3/Ad-FoxO3 cells showed significantly reduced phosphorylated Smad3 in the cytoplasm (Figure 6M) and significantly reduced total Smad3 in the nucleus (Figure 6P) compared to the Ad-Smad3 groups alone. Lastly, under these growth conditions, Ad-FoxO3-infected cells showed significantly increased cytoplasmic phosphorylated FoxO3 (Figure 6Q) with a trend (*p* = .07) for increased cytoplasmic total FoxO3 (Figure 6R) compared to Ad-GFP controls, and these increases were largely reversed with concomitant Ad-Smad3/Ad-FoxO3 infection. No significant changes in phosphorylated or total FoxO3 expression were detected in the nucleus across all Ad-groups (Figures 6S, T). Again, absolute levels of phosphorylated FoxO3 in the nucleus (Figure 6S) were extremely low compared to absolute levels of phosphorylated or total cytoplasmic FoxO3 (Figures 6Q, R) or total nuclear FoxO3 (Figure 6T).

### 3.6 Smad3 and FoxO3 control of E3 ubiquitin ligases and cytostatic p21/p27

**FIGURE 7:**
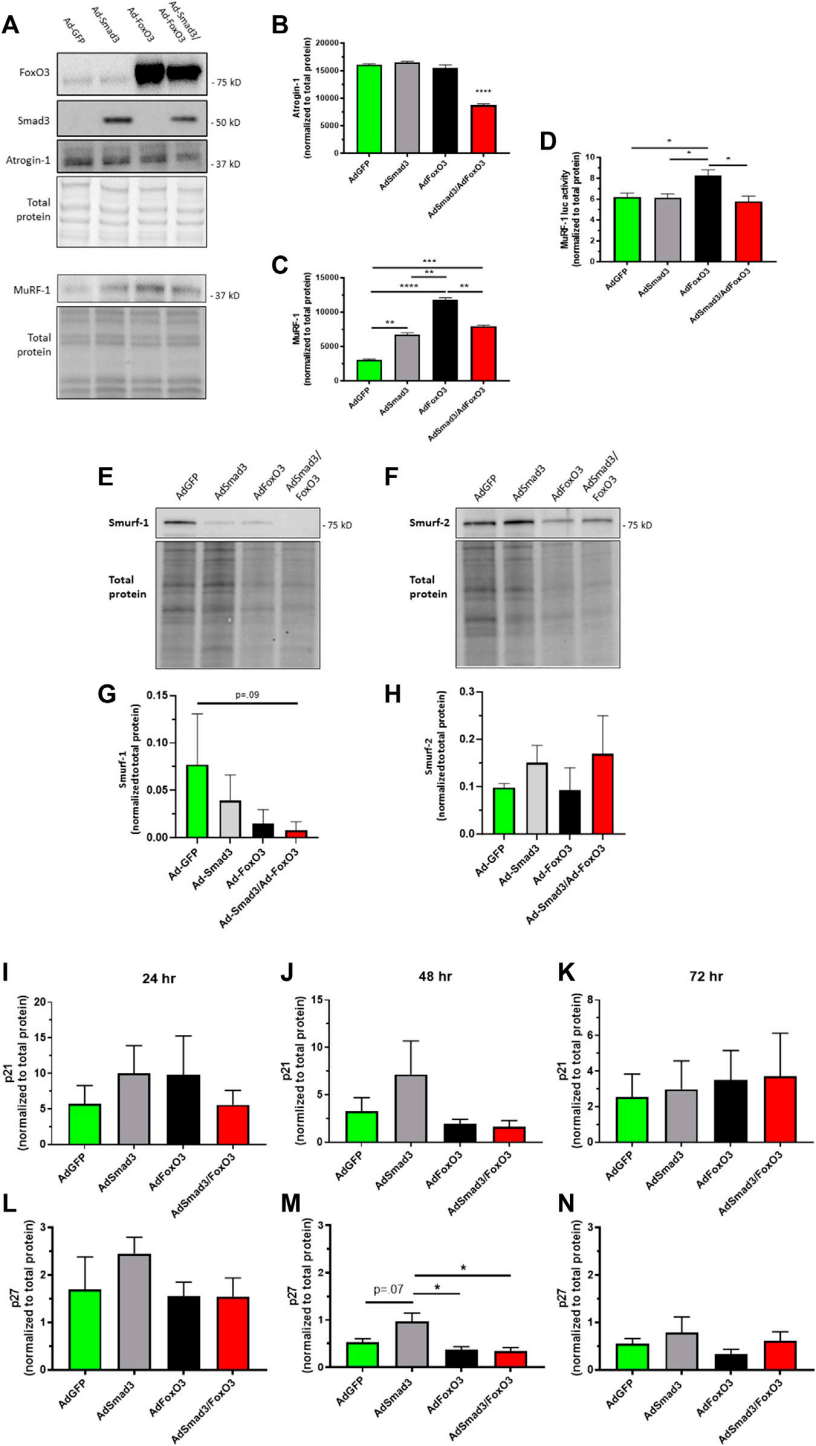
*Smad3 & FoxO3 control of E3 ubiquitin ligases & p21/p27* Rat ASM cells were infected for 24 h with Ad-GFP, Ad-Smad3, Ad-FoxO3, or Ad-Smad3/Ad-FoxO3, after which cell homogenates were probed for the E3 ubiquitin ligases Atrogin-1, MuRF-1, Smurf-1, and Smurf-2 as well as for the growth inhibitors p21 and p27. **(A)** Representative Western blots for Atrogin-1 and MuRF-1 along with Smad3 and FoxO3 and total protein blot from the same gels are shown. Interestingly, densitometry shows a significant decrease in Atrogin-1 in the Ad-Smad3/Ad-FoxO3 OE cells compared to all other groups **(B)**. Significant increases in MuRF-1 expression in Ad-Smad3, Ad-FoxO3, and Ad-Smad3/Ad-FoxO3 cells compared to Ad-GFP cells were observed, with the Ad-Smad3/Ad-FoxO3 cohort significantly lower than the Ad-FoxO3 group **(C)**. A MuRF-1 luciferase assay revealed a significant increase in MuRF-1 transcriptional activity in Ad-FoxO3 OE cells compared to all other Ad-groups including notably Ad-Smad3/Ad-FoxO3 OE cells **(D)**. Representative Western blots for Smurf-1 **(E)** and Smurf-2 **(F)** along with their corresponding total protein blots from the same gels, are shown. Densitometry **(G,H)** revealed no significant differences in Smurf-1 or Smurf-2 expression in any Ad-group, although a downward trend (overall *p* = 0.09) was detected for the Ad-Smad3/Ad-FoxO3 group versus the Ad-GFP cells for Smurf-1 **(G)**. Densitometry from Western blots for the cyclin-dependent kinase inhibitors p21 **(I–K)** and p27 **(L–N)** (normalized to total protein) 24, 48, and 72 h post-Ad-infection. No significant differences were detected in p21 across all Ad-groups at all time points **(I–K)**. After 48 h, expression of p27 was markedly elevated (*p* = 0.07) in Smad3 OE cells compared to GFP cells, and this elevation was significantly reduced in FoxO3 OE and Smad3/FoxO3 OE cells **(M)**. *n* = 3 independent experiments/group; *p < 0.05, **p < 0.01, ***p < 0.001, ****p < 0.0001 for comparisons as shown.

We then evaluated the impact of Ad- OE on the FoxO3-dependent E3 ubiquitin ligases Atrogin-1 and MuRF-1 and the Smad3-dependent E3 ubiquitin ligases Smurf-1 and Smurf-2 as these enzymes have been implicated in Smad3/FoxO3 signaling and assorted cellular processes including growth (Cohen et al., 2009; Paffett et al., 2012; Bollinger et al., 2014). Figure 7A shows a representative Western blot for the FoxO3 ubiquitin ligases Atrogin-1 and MuRF-1 including Smad3 and FoxO3 and total cellular protein after Ad-infection for 24 h. Densitometry showed a significant decrease in Atrogin-1 expression in the Ad-Smad3/Ad-FoxO3 OE cells compared to all other groups (Figure 7B). In comparison, Western analysis showed significant increases in MuRF-1 expression in Ad-Smad3, Ad-FoxO3, and Ad-Smad3/Ad-FoxO3 cells versus GFP cells, with the Ad-Smad3/Ad-FoxO3 cohort significantly lower than the Ad-FoxO3 group (Figure 7C). In complement, a MuRF-1 luc activity assay revealed a significant increase in MuRF-1 transcriptional activity in Ad-FoxO3 OE cells which was fully reversed in the Ad-Smad3/Ad-FoxO3 cells (Figure 7D). Representative Western blots for the Smad3 ubiquitin ligases Smurf-1 and Smurf-2 along with total cellular protein blots from the same gels after Ad-infection for 24 h are shown in Figures 7E, F. Densitometry failed to detect significant changes in Smurf-1 and Smurf-2 expression following Ad-Smad3, Ad-FoxO3, or Ad-Smad3/Ad-FoxO3 infection compared to GFP controls after 24 h, although a downward trend (*p* = 0.09) was detected for the Ad-Smad3/Ad-FoxO3 group versus the Ad-GFP cells for Smurf-1. In these Ad- OE cohorts, Western analysis was then performed for the cell cycle inhibitors p21 and p27. Densitometry (normalized to total cellular protein) at 24, 48, and 72 h post-Ad-infection failed to discern noticeable differences in p21 across all Ad-groups (Figures 7I–K). The same holds true for p27 after 24 and 72 h (Figures 7L, N), yet after 48 h, p27 expression was markedly elevated (*p* = 0.07) in Smad3 OE cells compared to GFP cells, and this increase was significantly reduced in the Ad-FoxO3 and Ad-Smad3/Ad-FoxO3 cells (Figure 7M).

### 3.7 Smad3 induces a growth phenotype in ASM cells which is normalized by FoxO3

**FIGURE 8:**
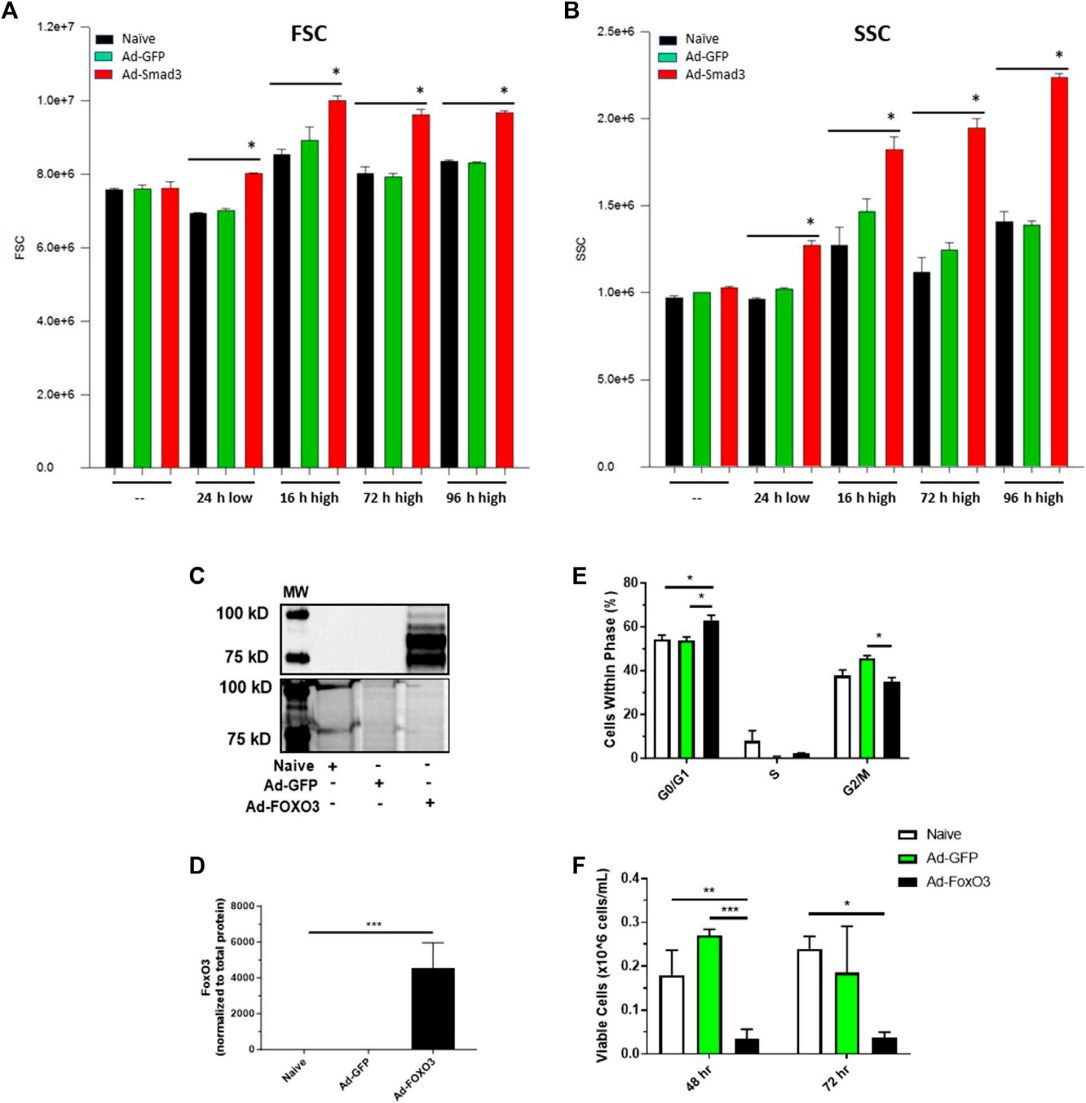
*Smad3 induces a growth phenotype while FoxO3 induces a growth arrested phenotype* Rat ASM cells were infected with Ad-GFP or Ad-Smad3 for 24 h in low serum media, after which cells were kept in low serum for 24 h or stimulated with high serum for between 16 and 96 h. Cells were then analyzed via flow cytometry for forward scatter (FSC) and side scatter (SSC), relative indicators of cell size and complexity/granularity consistent with enhanced growth. Seen in **(A)**, Smad3 OE significantly increased FSC after 24 h in low serum and consistently through 96 h in high serum compared to control naïve and GFP cells. Similarly, Smad3 OE significantly increased SSC after 24 h in low serum and consistently through 96 h in high serum compared to control cells **(B)**. Rat ASM cells were infected with Ad-GFP or Ad-FoxO3 for 24 h in low serum media, after which cells were homogenized and probed for FoxO3 protein expression or analyzed for cell growth. **(C)** A representative Western blot along with densitometry **(D)** for FoxO3 along with a total protein blot from the same gel from naïve, Ad-GFP, and Ad-FoxO3 OE cell homogenates. **(E)** Flow cytometry was performed to evaluate cell cycle progression in naïve, Ad-GFP, and Ad-FoxO3 cells after 24 h. FoxO3 OE cells demonstrate significant increases in quiescent G_0_/G_1_ and decreases in G_2_/M phases compared to control cells, suggestive of a growth restricted phenotype. **(F)** Ad-FoxO3 OE cells show significantly reduced viable cell numbers compared to naïve and Ad-GFP cells after 48 and 72 h. *n* = 3 independent experiments/group; *p < 0.05, **p < 0.01, ***p < 0.001 versus other groups as indicated.

We previously documented growth-promoting impact of Smad3 in rat ASM cells, from both broad TGF-β agonism and more precise Ad-Smad3 OE (Stone et al., 2015). Our observations revealed that Ad-Smad3 significantly increased percentage of cells in G_2_/M and increased absolute and viable cell numbers compared to naïve control and Ad-GFP cells. In new experiments, we verified the growth-promoting influence of Ad-Smad3 OE in rat primary ASM cells and determined the growth regulatory impact of FoxO3 OE alone or in conjunction with Smad3. Using flow cytometry, we observed that Ad-Smad3 OE significantly and consistently increased forward scatter (FSC) and side scatter (SSC), respective indicators of cell diameter/size and cell granularity/internal complexity and both supportive of a growth phenotype, compared to naïve and Ad-GFP cells through 96 h (Figures 8A, B). Using a crystal violet attachment assay, we observed Smad3 OE significantly increased ASM cell-substrate adhesion, another marker of the growth phenotype, compared to naïve and Ad-GFP cells over 72 h (data not shown). In a separate set of experiments with a focus on FoxO3, we first verified FoxO3 OE following Ad-FoxO3 infection (Figures 8C, D). We then used Ad-FoxO3 OE and observed cell cycle stagnation and cytostasis, with significantly increased cell numbers in quiescent G_0_/G_1_ and significantly decreased cell numbers in G_2_/M after 24 h (Figure 8E), as well as significantly reduced viable cell numbers after both 48 and 72 h (Figure 8F) compared to naïve and Ad-GFP cells.

**FIGURE 9:**
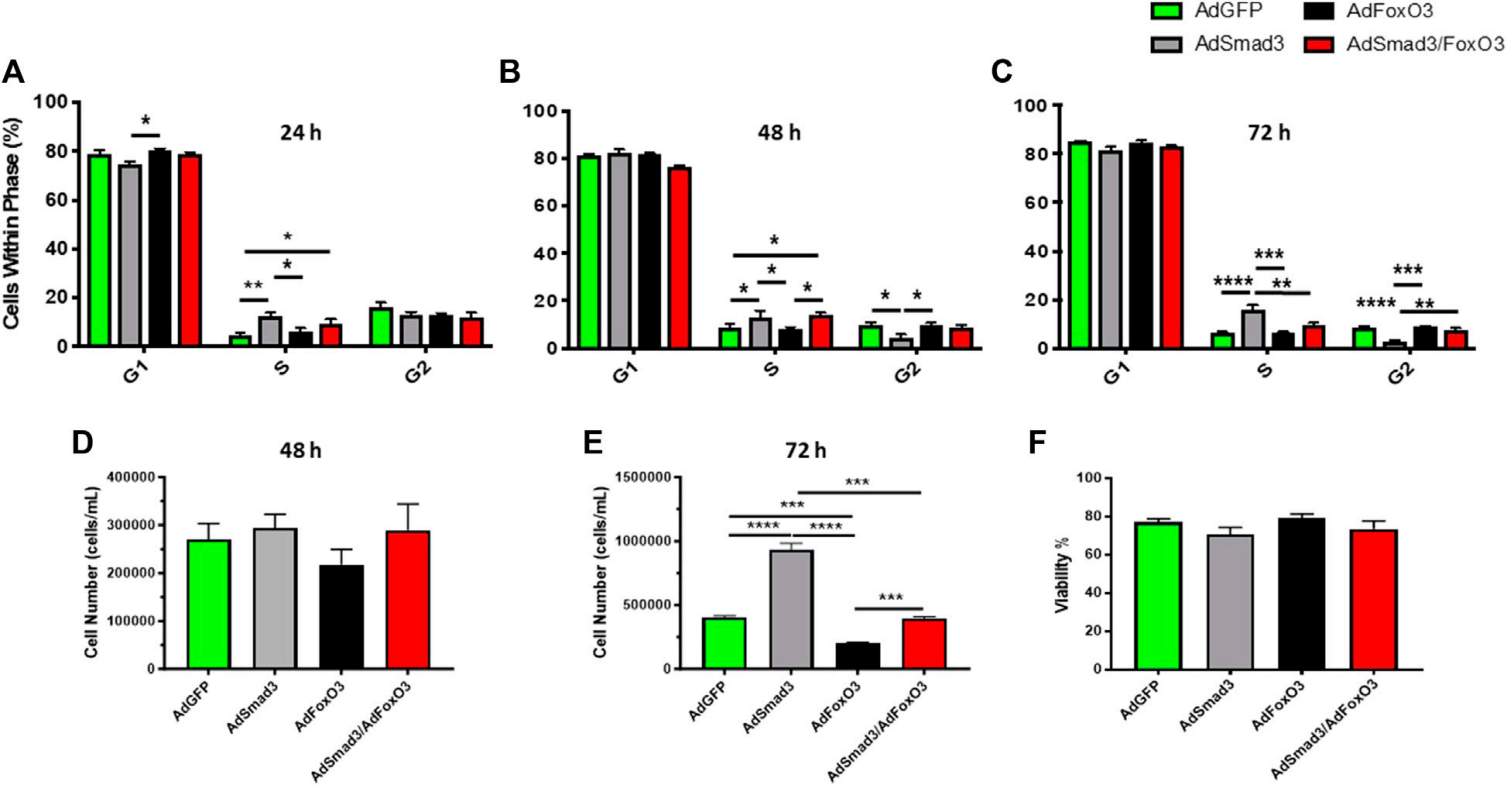
*FoxO3 normalizes Smad3-induced ASM cell growth* Rat ASM cells were infected for 24 h with Ad-GFP, Ad-Smad3, Ad-FoxO3, or Ad-Smad3/Ad-FoxO3, after which cell cycle progression, proliferation, and viability experiments were conducted over 72 h. Ad-Smad3 OE significantly increased DNA synthesis and cell cycle progression through 72 h **(A–C)**. While no changes in viable cell numbers were observed after 48 h **(D)**, after 72 h Ad-Smad3 OE increased viable cell numbers **(E)** compared to Ad-GFP cells. Ad-FoxO3 cells showed reduced numbers after 72 h compared to Ad-GFP controls **(E)**. Concomitant Ad-Smad3/Ad-FoxO3 OE normalized Smad3-stimulated cell cycle progression **(A–C)** and cell proliferation **(E)** compared to Ad-Smad3 cells at 72 h, and likewise, Ad-Smad3/Ad-FoxO3 OE reversed FoxO3-mediated reductions in cell cycle progression **(A–C)** and proliferation **(E)** compared to Ad-FoxO3. Lastly, no significant changes in cell viability were observed across all Ad-cohorts at 24, 48, or 72 h post-infection (48-h data shown in **(F)**. *n* = 4 independent experiments/group; *p < 0.05, **p < 0.01, ***p < 0.001, ****p < 0.0001 for comparisons as shown.

Lastly, cell cycle progression, proliferation, and viability experiments were conducted after 24, 48, and 72 h of Ad-OE for all cohorts (Figure 9). Consistent with earlier observations, Ad-Smad3 OE significantly increased cell cycle progression over 72 h (Figures 9A–C), with corresponding increases in viable cell numbers after 72 h (Figure 9E), compared to Ad-GFP cells. Also in agreement with earlier findings, Ad-FoxO3 cells showed significantly reduced numbers after 72 h compared to Ad-GFP controls (Figure 9E). Further, concomitant Ad-Smad3/Ad-FoxO3 OE normalized Smad3-stimulated cell cycle progression as well as FoxO3-mediated cytostasis (Figures 9A–C) and cell proliferation (Figure 9E) compared to respective Ad-Smad3 or Ad-FoxO3 cells over 72 h. Lastly, no significant changes in cell viability were observed across all Ad-cohorts at 24-, 48-, or 72-h post-infection (48-h time point data shown in Figure 9F).

## 4 Discussion

As a central pillar in the pathogenesis of CVD, conversion of contractile, quiescent ASM cells to a synthetic, proliferative state encourages aberrant vessel growth and contributes to clinical repercussions of CVD (Holt et al., 2016; Holland et al., 2018; American Heart Association, 2023; Williams et al., 2023). A plethora of transcriptional, translational, and post-translational factors with capacity to regulate ASM growth has been identified, and of these, transcriptional Smad3 and FoxO3 are gaining promise due in part to their unique relationship (Gomis et al., 2006; Bollinger et al., 2014) and discrete yet opposing capacities to control tissue growth (Fu and Peng, 2011; Stone et al., 2015). However, the nature of a Smad3/FoxO3 nexus in ASM including dependency on phosphorylation status and ability to regulate cell growth is not clear. The purpose of this study was to determine that a coordinated, phosphorylation-specific relationship exists between Smad3 and FoxO3 in the control of ASM cell growth, and we hypothesized that FoxO3 acts to normalize Smad3-driven ASM growth. In primary ASM cells using quiescent or growth conditions and Ad-mediated OE, key findings reveal unique cellular distribution of Smad3 and FoxO3 in growth; Smad3 OE increases Smad3 expression which is reversed with concomitant FoxO3 OE; FoxO3 OE increases FoxO3 expression which is reduced with simultaneous Smad3 OE; FoxO3 OE induces the ubiquitin ligase MuRF-1 which is reversed with concomitant Smad3 OE; and Smad3 OE stimulates growth while FoxO3 OE inhibits growth. A major, therapeutically important conclusion from these findings is the ability of FoxO3 to normalize Smad3-induced growth in ASM cells. Results also support capacity of Smad3 to serve mitigating roles on FoxO3-induced cytostasis, with all effects dependent on discrete phosphorylation states and cellular localization and involving MuRF-1. These observations also provide evidence for potential therapeutic use of Smad3 and/or FoxO3 as targets for ASM growth control in the context of CVD.

Our previous observations showing induction of the pleiotropic chemokine TGF-β1 following injury in rat carotid arteries (Tulis et al., 2005) prompted us to dig deeper into potential growth-regulatory roles of TGF-β1 and its primary intracellular effector, Smad3. Our follow-up study (Stone et al., 2015) used rat commercial A7R5 and rat primary ASM cells with recombinant TGF-β1 induction as well as Ad-Smad3 OE, an approach used to bypass potential side effects of wide-ranging TGF-β1 activation. We found that Smad3 stimulated cell cycle progression and increased cell numbers compared to vehicle or Ad-GFP controls due in part to increased cyclin D/E and CDK2/4 and reduced cytostatic p21 and p27. In the current study, our first experiments verified Smad3 induction in an ASM growth-provoking rat carotid artery injury model (Tulis, 2007a; Tulis, 2007b; Joshi et al., 2011; Holt and Tulis, 2013), confirming our previous *in vivo* observations and *in vivo* findings from other investigators (Tsai et al., 2009) and lending credence for more focused and controlled study of the mechanisms of Smad3 in primary ASM cells. It is important to mention that our *in vivo* data revealed that phosphorylated and total Smad3 were both significantly upregulated following intact arterial injury (Figures 1B, D), yet considering that the increase in total Smad3 surpassed the increase in phosphorylated Smad3, a significantly reduced ratio was observed (Figure 1E). We have previously documented the biological significance of alterations in solitary phosphorylated proteins as well as in solitary total proteins and both the utility and caveats of using a phosphorylated to total protein ratio as a relative indicator of protein activity (Mendelev et al., 2009; Joshi et al., 2011; Adderley et al., 2012; Holt et al., 2016; Williams et al., 2023). Thus, one must consider both readouts of individual phosphorylated protein and total protein as well as their ratio for full understanding of their biological impact. Lastly, in these *in vivo* experiments we failed to detect significant alterations in whole vessel FoxO3 with injury (Figures 1F–H). One must remember that under these *in vivo* conditions, contributions from associated cell types may interfere with observed results. For example, FoxO3 can be synthesized by immune cells (Hartwig et al., 2021) and fibroblasts (Nho et al., 2011), both involved in the injury response, in addition to medial wall ASM cells, likely confounding our *in vivo* observations. Thus, to achieve a more pointed focus on Smad3 and FoxO3 signaling in growth, our emphasis for the remainder of this study focused on the homogenous, clonal *in vitro* population of primary ASM cells.

Alongside Smad3, the FoxO family of transcription factors has been widely investigated for roles in various cellular processes including differentiation, apoptosis, matrix degradation, and oxidative stress responses (van der Horst and Burgering, 2007; Benayoun et al., 2011; Yu et al., 2018). Further, generic FoxO signaling has been shown to inhibit proliferation via elevated p21 and p27 in commercial human ASM cells (Abid et al., 2005; Mahajan et al., 2012). However, impact of the FoxO3 during ASM growth is not clear. Also, it has been suggested that a cooperative, synergistic relationship exists between Smad3 and FoxO3 transcription factors in other cell types (Gomis et al., 2006; Bollinger et al., 2014), although the nature of their relationship and growth-regulatory capacities in ASM is not known. With this in mind, we examined cellular localization of individual Smad3 and FoxO3 in quiescent (low serum) or growth (high serum) media to determine their cellular distribution.

Central to our experimental approach, we first wanted to validate our use of low serum (0.2%) media and high serum (10%) media as respective *in vitro* models of quiescent and growth states. Rat ASM cells grown in high serum media had markedly (*p* = 0.1) elevated viable cell numbers after 24 h and significantly increased viable numbers after 48 h compared to cells grown in low serum (Figure 2A). Next, low and high serum-treated cell homogenates were probed for Erk1/2 (p42/p44 MAPK), an established mitogen and inducer of wide-ranging aspects of cellular growth including migration, differentiation, metabolism, and proliferation (Roskoski Jr, 2012; Suwanabol et al., 2012). The phosphorylated Erk1/2 to total Erk1/2 ratio was significantly increased in cells in high serum compared to those in low serum after 24 h, with a subsequent reduction after 48 h, consistent with the timing of Erk1/2 activation during the cell cycle (Roberts et al., 2002) and also consistent with the impact of inactivating phosphatases (Roskoski Jr, 2012; Stone et al., 2012; Stone et al., 2013). Lastly, given the well-documented phenotypic switching of ASM cells during growth, involving both loss of contractile elements and increased proliferative/synthetic elements (Thyberg et al., 1990; Beamish et al., 2010; Holt et al., 2016; Holland et al., 2018; Williams et al., 2023), we evaluated expression of PKG-1, an established marker of phenotypic switching, in ASM cells incubated in low or high serum media for 48 h (Figure 2C). Consistent with our recent observations in growth-stimulated rat ASM cells compared to those under quiescent conditions (Williams et al., 2023), new observations reveal that cells in high serum show significantly reduced PKG-1 expression compared to those grown in low serum media, further validating our use of low serum and high serum media as representative models of quiescent and growth states, respectively.

In our low serum and high serum models, confocal photomicrographs of adherent ASM cells grown for 24 h and immunostained for Smad3 and FoxO3 are shown in Figure 3A. Image densitometry of cytoplasmic versus nuclear expression provided initial evidence for increased nuclear Smad3, decreased cytoplasmic Smad3, and increased cytoplasmic FoxO3 in high serum, growth conditions compared to quiescent conditions (Figures 3B, C). One must keep in mind the relative subjectivity and semi-quantitative nature of image-based densitometry. In turn, we prepared cytoplasmic and nuclear cell fractions of quiescent (low serum) and growing (high serum) cells, validated them using established cytoplasmic and nuclear markers, and probed them for Smad3 and FoxO3 expression in a much more objective and quantifiable approach. Seen in Figure 3D, expression of lamin A, a mammalian protein precisely localized to the cell nuclear lamina (Dubik and Mai, 2020), was significantly elevated in our nuclear fractions under both low and high serum conditions compared to our cytoplasmic fractions. Conversely, GAPDH, a glycolytic enzyme predominantly localized to the cytosol (Zhang et al., 2015), was significantly expressed in our cytoplasmic fractions in low serum, and markedly (*p* = 0.06) expressed in the cytoplasm in high serum, compared to the nuclear fractions (Figure 3E). We then used Western evaluation of Smad3 and FoxO3 in ASM cytoplasmic and nuclear fractions under these conditions (Figures 3F–I). Densitometry of these blots revealed that cells in high serum media showed significantly higher cytoplasmic and nuclear Smad3 and predominantly cytosolic FoxO3 compared to cells in low serum media (Figure 3J), each normalized to its respective total cytoplasmic protein or total nuclear protein. These observations show that serum-induced proliferation is sufficient to uniquely distribute Smad3 and FoxO3 in the cell, activating Smad3 in the nucleus and inactivating FoxO3 in the cytosol. These findings are also consistent with earlier results from our lab (Stone et al., 2015) and others (Abid et al., 2005; Lee et al., 2007; Tsai et al., 2009; Mahajan et al., 2012) showing growth promotion by singular Smad3 and growth inhibition by singular FoxO3 in ASM cells.

Next, considering these preferential intracellular expression patterns for Smad3 and FoxO3 in quiescent versus growth conditions, we used Ad-mediated gene delivery to discern influence of Smad3 and/or FoxO3 OE on cytoplasmic and nuclear expression of Smad3 and FoxO3. For validation of our Ad-delivery approach, Western analysis on Ad-infected ASM cell lysates 24 h post-infection confirms robust Smad3 expression in the Smad3 OE cells, and robust Smad3 expression in the Smad3 OE cells, normalized to total cellular protein (Figure 4A). Further, phase contrast and GFP fluorescent photomicrographs of Ad-infected ASM cells after 72 h are shown (Figure 4B), revealing consistent GFP expression in all GFP-encoding cells (Ad-Smad3 does not contain an IRES and does not encode GFP). Next, cell fractions were prepared after 48 h of Ad- OE, and representative Western blots for cytoplasmic and nuclear Smad3 and FoxO3 are shown (Figures 4C, D). Densitometry reveals significant cytoplasmic Smad3 induction in Ad-Smad3 cells, which was reversed in Ad-Smad3/Ad-FoxO3 cells (Figure 4E), and similarly induction of cytoplasmic FoxO3 and nuclear FoxO3 in Ad-FoxO3 cells were reduced in the Ad-Smad3/Ad-FoxO3 groups (Figures 4G, H). A key takeaway from these data is the almost complete reversal of Ad-mediated Smad3 and FoxO3 induction, respectively, in the presence of combined Ad-Smad3/Ad-FoxO3 OE. These findings argue for an antagonistic relationship with reciprocal negative cooperativity between transcriptional Smad3 and FoxO3 in ASM cells; yet, the essentiality of phosphorylation in determining Smad3 and FoxO3 cellular localization and transcriptional activation particularly during growth directed us to more in-depth analyses.

Smad3 and FoxO3 have opposing mechanisms of phosphorylation-dependent transcriptional activation: Smad3 phosphorylation at Ser_423/425_ leads to nuclear localization and transcriptional activation, generally considered synthetic and proliferative (Shi and Massague, 2003; Tsai et al., 2009; Stone et al., 2015; Holland et al., 2018), while FoxO3 phosphorylation at the preferred Akt site Thr_32_ results in either cytosolic retention or nuclear export and cytoplasmic localization (Yang and Hung, 2009; Fu and Peng, 2011; Deng et al., 2015). Logically, unphosphorylated FoxO3 is the active form and is localized to the nucleus, where it can maintain levels of cytostatic p21 and/or p27 (Mahajan et al., 2012; Tucka et al., 2014) and provoke apoptosis (Skurk et al., 2004). Thus, we believe our proposed inhibitory relationship between Smad3 and FoxO3 would become apparent following global Ser/Thr phosphorylation by enhanced pro-growth aspects of Smad3 with parallel constraints on the growth protective actions of FoxO3. First, we evaluated the impact of Ad- OE on both total and phosphorylated Smad3 and FoxO3 in ASM cell cytoplasmic and nuclear fractions under quiescent, low serum conditions for 48 h (Figures 5A–J) or 72 h (Figures 5K–T). General findings from these experiments conducted in quiescent media show that Ad-Smad3 OE induced both phosphorylated and total Smad3 in the cytoplasm and nucleus, all reversed with concomitant Ad-FoxO3 OE. Further, in these conditions, Ad-FoxO3 OE increased both phosphorylated and total FoxO3 in the cytoplasm as well as total FoxO3 in the nucleus, all reversed with Ad-Smad3 OE. Of note, nuclear phosphorylated FoxO3 was not markedly impacted by Ad- OE within any group, and additionally, absolute levels of phosphorylated FoxO3 in the nucleus were negligible compared to absolute levels of total FoxO3 in the nucleus or total or phosphorylated FoxO3 in the cytoplasm. This is likely due to phosphorylation-dependent cytosolic localization of phosphorylated FoxO3. Next, following the same experimental approach, we compared these observations to those occurring during growth using high serum media after 48 h (Figures 6A–J) or 72 h (Figures 6K–T). Consistently, in general Smad3 induction from Ad-Smad3 OE was largely reversed in the presence of Ad-FoxO3 OE, and likewise, FoxO3 induction from Ad-FoxO3 OE was largely reversed with concomitant Ad-Smad3 OE. Again, absolute levels of phosphorylated FoxO3 in the nucleus were extremely low compared to FoxO3 expression elsewhere, likely due to nuclear export and cytoplasmic localization of phosphorylated FoxO3. Our observations using Ad-mediated OE of Smad3 and/or FoxO3 are consistent with reported cellular trafficking of transcription factors including Smad3 and FoxO3 between the nucleus and cytoplasm or other cellular compartments (Liu et al., 2018; Rozes-Salvador et al., 2018; Fasano et al., 2019), which then drives their respective transcriptional growth-regulating capacities.

Ubiquitin ligases in the ubiquitin-proteosome system (UPS) are essential for homeostatic protein degradation and elimination as our group (Cohen et al., 2009; Davis et al., 2020) and others (Demasi and Laurindo, 2012) have detailed. As such, the UPS has critical roles in regulating assorted cellular processes such as signal transduction, differentiation, proliferation, and apoptosis. In the current study we analyzed the impact of Ad- OE on the FoxO3-dependent ubiquitin ligases Atrogin-1 and MuRF-1, and on the Smad3-dependent ubiquitin ligases Smurf-1 and Smurf-2, key E3 UPS enzymes implicated in Smad3/FoxO3 signaling and related cellular activities including growth (Sandri et al., 2004; Cohen et al., 2009; Demasi and Laurindo, 2012; Paffett et al., 2012; Bollinger et al., 2014). Representative Western blots are shown for FoxO3, Smad3, Atrogin-1, and MuRF-1 in Figure 7A. Expression of Atrogin-1, a FoxO3-driven ubiquitin ligase (Sandri et al., 2004), was significantly reduced in the presence of combined Ad-Smad3/Ad-FoxO3 compared to all other Ad- OE cohorts (Figure 7B). Despite Atrogin-1 being a FoxO3-controlled ligase, seeing that Atrogin-1 was decreased only in the Ad-Smad3/Ad-FoxO3 cohort does not support its role in Smad/FoxO growth control observed in this study. In comparison, MuRF-1, another FoxO3-specific ubiquitin ligase (Cohen et al., 2009; Bollinger et al., 2014), was significantly increased in both expression and activity with Ad-FoxO3 OE compared to GFP controls (Figures 7C, D). Further, this FoxO3-induced stimulation of MuRF-1 was completely reversed with Ad-Smad3 co-infection. MuRF-1 is widely expressed in cardiac and skeletal muscle (Cohen et al., 2009; Bollinger et al., 2014), especially during periods of rapid protein turnover (Bodine et al., 2001), but other than involvement in *postpartum* involution of uterine SM (Bdolah et al., 2007), the role of MuRF-1 in SM is not known. Theoretically, given its capacity to degrade cytoskeletal proteins in these other cell types (Cohen et al., 2009), in ASM MuRF-1 may operate as a protective measure to limit aberrant growth inherent in vascular injury or disease. Given the central roles of Smad3 and FoxO3 in ASM growth, MuRF-1 may represent a novel therapeutic target that works to combat abnormal ASM growth through moderation of key cytoskeletal elements, consistent with the roles of MuRF-1 in skeletal and cardiac muscle (Peris-Moreno et al., 2020). Further, considering reports of Smad-mediated Smurf upregulation (Zhang et al., 2001; Piersma et al., 2015; Koganti et al., 2018), with most findings implicating inhibitory Smad7 as the primary inducer of Smurf expression, in our study we failed to detect significant induction or suppression of either Smurf-1 or Smurf-2 following Ad-Smad3 OE (and all other Ad-groups) compared to Ad-GFP controls (Figures 7E–H). Lastly, among the many cell cycle regulatory factors that govern ASM cell growth, the cyclin-dependent kinase inhibitors p21 and p27 are of critical importance (Abid et al., 2005; Mahajan et al., 2012; Yu et al., 2018). Following our Ad- OE experimental design, Western analyses of p21 at 24, 48, and 72 h post-Ad- infection failed to discern significant differences across all Ad-groups (Figures 7I–K). In comparison, after 48 h, p27 expression was markedly elevated (*p* = 0.07) in Smad3 OE cells compared to GFP cells, and this elevation was significantly reduced in FoxO3 OE and Smad3/FoxO3 OE cells (Figure 7M). No detectable changes were otherwise observed for p27 at 24 or 72 h across all cohorts. This transient induction of p27 only at 48 h post-OE argued against continued efforts to analyze associated cyclins or cyclin-dependent kinases, as the expression of these cell cycle regulators is cyclical and largely time-dependent as we (Stone et al., 2013; Stone et al., 2015) and others (Roberts et al., 2002) have documented.

Lastly, we performed experiments to analyze the impact of concomitant Smad3/FoxO3 OE on cell cycle progression and proliferation. To extend our earlier findings showing that Ad-Smad3 OE provoked cell cycle progression and increased proliferation in rat primary ASM cells (Stone et al., 2015), new flow cytometry results reveal that Ad-Smad3 OE significantly increased flow cytometric FSC and SSC, respective indicators of cell diameter/size and cell granularity/internal complexity consistent with a growth phenotype, compared to control cells through 96 h (Figures 8A, B). We also observed that Smad3 OE significantly increased cell-substrate adhesion, another indicator of growth, compared to controls over 72 h (data not shown). These new observations combined with our previous results firmly establish Smad3 as a growth inducer in ASM cells. Next, in separate experiments we analyzed the impact of FoxO3 OE on ASM cell growth. Findings showed significantly increased cell numbers in quiescent G_0_/G_1_ with reduced cell numbers in G_2_/M phases after 24 h and reduced cell numbers after both 48 and 72 h in FoxO3 OE cells compared to control cells (Figures 8C–F). We then assessed the impact of combined OE of Smad3/FoxO3 on cell growth. Flow cytometry results (Figures 9A–C) across 72 h broadly support cell cycle stimulation by Smad3 and cell cycle stagnation by FoxO3. Further, after 72 h concomitant Smad3/FoxO3 OE fully reversed both the enhanced proliferation seen with Smad3 OE alone and the cytostatic effects observed with FoxO3 OE alone (Figure 9E). These results add additional support for a reciprocal, antagonistic relationship between transcriptional Smad3 and FoxO3 in governing ASM cell growth.

Activated Smad3, when complexed with receptor-regulated Smad2 and common Smad4 in the nucleus, is theorized to require association with other DNA-binding proteins to elicit genetic control (Gomis et al., 2006). One potential DNA binding partner is FoxO3, and in human commercial keratinocytes Smad3 and FoxO3 are synchronously regulated by a common upstream agonist, TGF-β, and thus Smad3/FoxO3 has been coined a “synexpression group” (Gomis et al., 2006). Earlier work by our group (Bollinger et al., 2014) in human embryonic kidney cells and mouse myoblasts identified cooperativity and synergism between Smad3 and FoxO3 in terms of MuRF-1 transcriptional activity, and from that study we concluded that Smad3 binding to DNA is required for optimal activation of closely located FoxO3 DNA binding sites in those cell/tissue types. In the current study it was surprising, that, when using the same MuRF-1 promoter luciferase reporter, results showed that Smad3 is inhibitory to FoxO3 in the control of MuRF1 transcriptional activity. This difference in tissue response may be due to interacting proteins that are unique to ASM that limit binding of Smad3 and/or FoxO3 to DNA when they are co-expressed or perhaps limit binding of transcriptional machinery to DNA when both Smad3 and FoxO3 are bound. Regardless, to our knowledge, this is the first report to characterize a Smad3/FoxO3 relationship in ASM, particularly as a regulatory underpinning of growth.

During our study, several limitations were realized that warrant brief mention. Female rats were not used to generate our primary cells due to potential impact of estrogen on ASM cell growth (Yuan et al., 2002); nevertheless, complete analysis of cellular growth mandates inclusion of both male and female cells to identify possible sex-dependent effects. Our Ad-experiments were designed in *a priori* fashion for specific OE of Smad3 and/or FoxO3 to avoid potential confounding influence of broad upstream agonism by TGF-β, Akt, or other stimuli. For full understanding of the regulatory roles of these transcriptional elements, one could use phosphorylation-deficient mutants in addition to phosphomimetics as we have detailed in ASM cells (Holt et al., 2016). Also, despite the precision of Ad-delivery of Smad3 and FoxO3 in the clonal homogenous setting of primary ASM cultures, the biological, whole-body actions of Smad3 and FoxO3 may suffer from interference by homologous elements such as Smad1/2/4 and/or FoxO1. Lastly, on the basis of our findings, several future directions for study could include use of bulk RNAseq to identify transcript presence and quantity, therefore indicating activation of these different signaling pathways, use of ChIP-Seq to discern common DNA binding sites for Smad3 and FoxO3, or use of image-derived densitometry to complement Western blot cell fraction data for cellular localization of Smad3/FoxO3, together gaining further insight into the mechanisms of Smad3 and FoxO3 and their unique relationship in ASM growth.

Collectively, these data largely support our original notions and provide evidence that a reciprocal and antagonistic relationship exists between the transcription factors Smad3 and FoxO3 in ASM cells. Our findings confirm Smad3 as a growth promoter and FoxO3 as a cytostatic growth inhibitor in rat primary ASM cells. Further, using target-specific Ad- OE, observations revealed capacity of FoxO3 to reverse growth-stimulating effects of Smad3, and conversely, of Smad3 to reverse FoxO3 actions. Evidence is also provided for the ubiquitin ligase MuRF-1 in this mutual Smad3/FoxO3 growth control in ASM cells. These new findings shed light on Smad3/FoxO3 control of ASM cell growth and offer evidence for their potential utility as therapeutic targets to combat CVD.

## Data Availability

The original contributions presented in the study are included in the article, and further inquiries can be directed to the corresponding author.
